# Blockade of IL‐33 signalling attenuates osteoarthritis

**DOI:** 10.1002/cti2.1187

**Published:** 2020-10-23

**Authors:** Zengliang He, Yan Song, Yongxiang Yi, Fengzhuo Qiu, Junhua Wang, Junwei Li, Qingwen Jin, Pradeep Kumar Sacitharan

**Affiliations:** ^1^ Department of Orthopedics The Second Hospital of Nanjing The Affiliated Hospital of Nanjing University of Chinese Medicine Nanjing China; ^2^ Department of General Surgery The Second Hospital of Nanjing The Affiliated Hospital of Nanjing University of Chinese Medicine Nanjing China; ^3^ Department of Neurology The Sir Run Run Hospital Nanjing Medical University Nanjing China; ^4^ College of Veterinary Medicine Qingdao Agricultural University Qingdao China; ^5^ The Institute of Ageing and Chronic Disease University of Liverpool Liverpool UK; ^6^ Department of Biological Sciences Xi'an Jiaotong‐Liverpool University Suzhou Industrial Park Suzhou China

**Keywords:** chondrocytes, IL‐33, inflammation, osteoarthritis, ST2

## Abstract

**Objectives:**

Osteoarthritis (OA) is the most common form of arthritis characterised by cartilage degradation, synovitis and pain. Disease modifying treatments for OA are not available. The critical unmet need is to find therapeutic targets to reduce both disease progression and pain. The cytokine IL‐33 and its receptor ST2 have been shown to play a role in immune and inflammatory diseases, but their role in osteoarthritis is unknown.

**Methods:**

Non‐OA and OA human chondrocytes samples were examined for IL‐33 and ST2 expression. Novel inducible cartilage specific knockout mice (IL‐33^Acan CreERT2^) and inducible fibroblast‐like synoviocyte knockout mice (IL‐33^Col1a2 CreERT2^) were generated and subjected to an experimental OA model. In addition, wild‐type mice were intra‐articularly administered with either IL‐33‐ or ST2‐neutralising antibodies during experimental OA studies.

**Results:**

IL‐33 and its receptor ST2 have increased expression in OA patients and a murine disease model. Administering recombinant IL‐33 increased OA and pain *in vivo*. Synovial fibroblast‐specific deletion of IL‐33 decreased synovitis but did not impact disease outcomes, whilst cartilage‐specific deletion of IL‐33 improved disease outcomes *in vivo*. Blocking IL‐33 signalling also reduced the release of cartilage‐degrading enzymes in human and mouse chondrocytes. Most importantly, we show the use of monoclonal antibodies against IL‐33 and ST2 attenuates both OA and pain *in vivo*.

**Conclusion:**

Overall, our data reveal blockade of IL‐33 signalling as a viable therapeutic target for OA.

## Graphical abstract

In this study, we found that expression of IL‐33 and ST2 is increased in human and murine osteoarthritis (OA) samples. We also demonstrate that the main sources of IL‐33 during OA are chondrocytes and not synovial fibroblasts. Most importantly, neutralising IL‐33 and ST2 attenuates OA and pain.

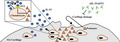

## Introduction

Osteoarthritis (OA) is the most common form of arthritis.^1^ There are no effective therapeutic options for OA, and joint replacement still remains the only approach to treat the disease.^1^ There is an urgent need to find suitable drug targets to address both disease pathogenesis and pain. Increased joint inflammation caused by cytokines such as IL‐1 and TNF‐α has been shown to drive OA pathogenesis.^2^, ^3^ These cytokines increase the production of proteases by chondrocytes, the only resident cells in cartilage, to cause cartilage degradation and secondary disease symptoms such as pain.^4^ Monoclonal antibodies targeting IL‐1 and TNF‐α and their respective receptors have showed poor efficacy as OA therapies.^5^, ^6^ Hence, other key cytokines responsible for OA pathogenesis that may be effective drug targets still remain elusive.

IL‐33, a member of the IL‐1 family, plays a role in both innate and adaptive immunity.^7^ IL‐33 exerts its activity by binding to its specific primary receptor ST2.^8^ Targeting both IL‐33 and ST2 demonstrated protective effects in respiratory diseases,^9^, ^10^, ^11^ skin diseases,^12^, ^13^ kidney disease^14^, ^15^ and neurological autoimmune pathologies.^16^, ^17^ IL‐33 and ST2 concentrations were also found to be increased in sera and synovial fluid of rheumatoid arthritis (RA) patients.^18^, ^19^ Blocking ST2 improved experimental RA.^20^ However, the role of IL‐33 in OA is unknown. Using human and murine models, we investigate whether IL‐33 signalling is involved in OA pathogenesis and the potential for targeting the cytokines signalling pharmacologically. In this study, we show IL‐33 and ST2 levels are increased in OA patients. For the first time, we demonstrate chondrocyte‐specific IL‐33 production not synovial fibroblast‐specific IL‐33 production is responsible for OA pathogenesis *in vivo*. Finally, we demonstrate blocking IL‐33 signalling reduces the production of proteases by human and murine chondrocytes and attenuates OA and pain *in vivo*.

## Results

### Increased levels of IL‐33 and ST2 in human and murine OA

**Figure 1:**
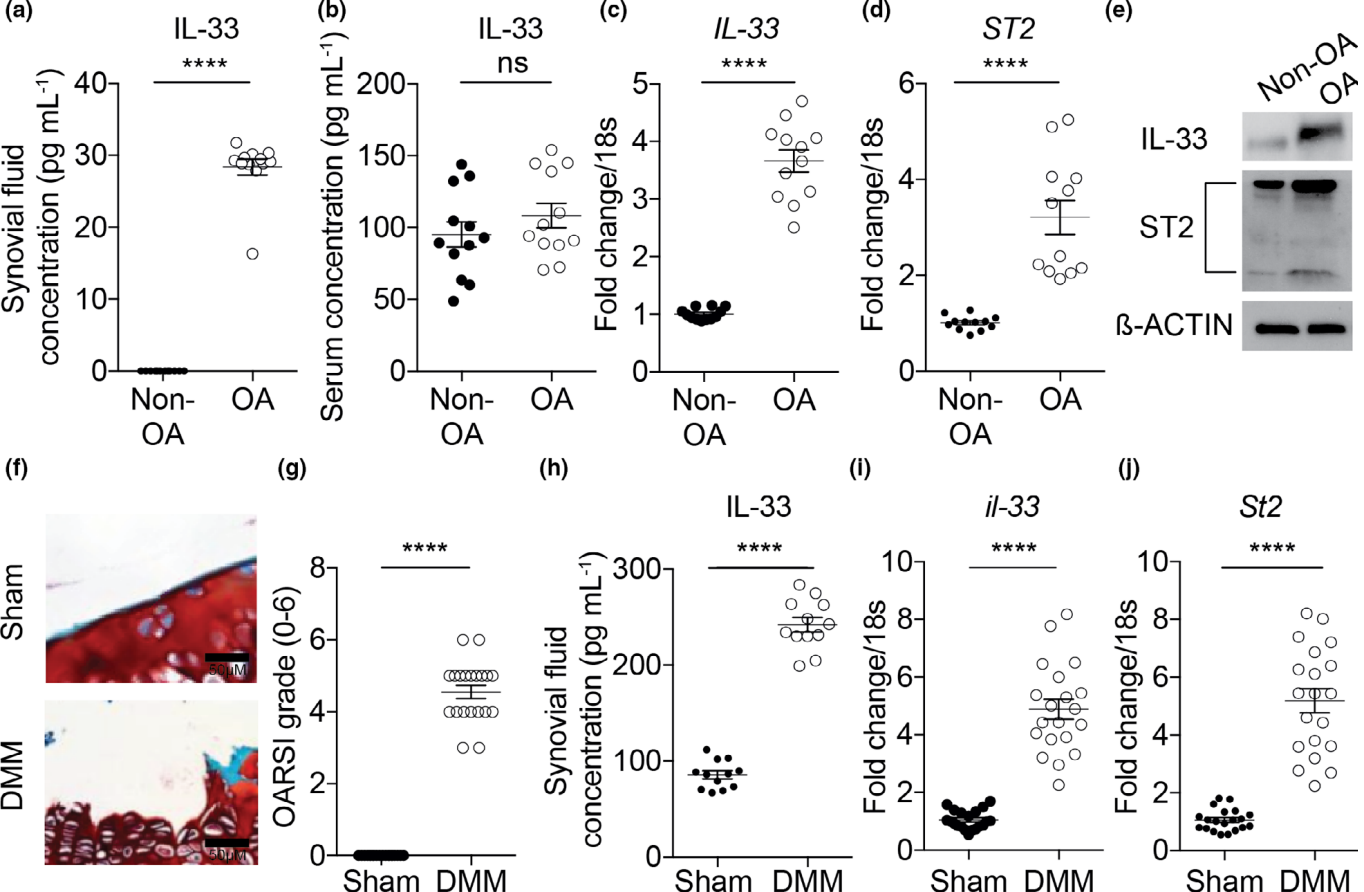
Increased levels of IL‐33 and ST2 in human and murine OA. IL‐33 concentration (pg mL^−1^) in **(a)** SF and **(b)** serum of non‐OA (*n* = 12) and OA patients (*n* = 12). **(c, d)** mRNA and (**e)** protein expression of IL‐33 and ST2 in isolated human chondrocytes from non‐OA (*n* = 12) and OA patients (*n* = 12). **(f, g)** OARSI scoring of cartilage tissue of sham‐operated (*n* = 20) or DMM‐operated (*n* = 20) WT mice (12 weeks post‐surgery end timepoint). **(h)** IL‐33 concentration (pg mL^−1^) in SF of sham‐operated (*n* = 12) or DMM‐operated (*n* = 12) WT mice (12 weeks post‐surgery end timepoint). **(i, j)** mRNA expression of IL‐33 and ST2 in whole knee joints from sham‐operated (*n* = 20) or DMM‐operated (*n* = 20) WT mice (12 weeks post‐surgery end timepoint). All RT‐qPCR gene expressions were normalised to the endogenous level of 18 s in respective groups. Data are expressed as mean ± S.E.M. with unpaired 2‐tailed Student’s *t*‐tests. *n* indicates the number of human specimens or mice per group. NS = non‐significant. *P* < 0.0001 is represented as ****.

We examined patient serum and SF to investigate whether IL‐33 signalling is involved in OA. IL‐33 concentration was increased in SF, but not in sera, of OA patients compared to non‐OA patients (Figure 1a and b). We also observed increased mRNA and protein expression of IL‐33 and ST2 in isolated chondrocytes from OA patients compared to non‐OA patients (Figure 1c–e). We went on to investigate whether IL‐33 and ST2 levels are elevated in murine OA (Figure 1f, g and Supplementary figure 1a, b). Previous gene expression studies did not show any change in IL‐33 or ST2 gene expression at early time points post‐surgery in mouse models of OA.^21^, ^22^, ^23^ Hence, we used a later time point of 12 weeks post‐DMM surgery for our end‐point readouts. IL‐33 concentration was increased in SF, but not serum, of mice with confirmed OA (Figure 1h and Supplementary figure 1c). In addition, expression of IL‐33 and ST2 was increased in knee joints of mice with OA compared to mice without OA (Figure 1i and j and Supplementary figure 1d).

### Increasing IL‐33 levels exacerbates OA *in vivo*

**Figure 2:**
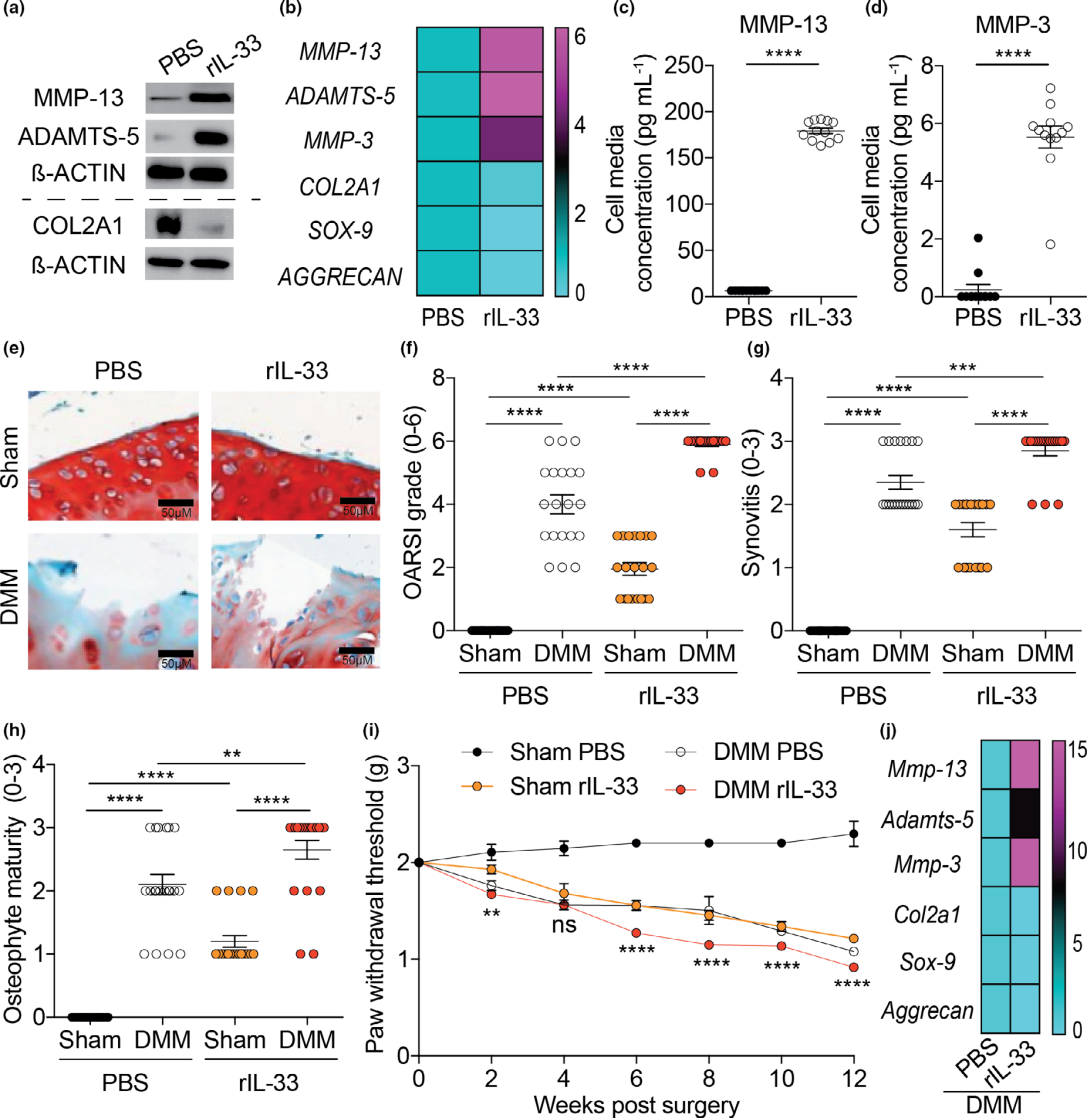
Increasing IL‐33 levels exacerbates OA *in vivo*. **(a)** protein and **(b)** mRNA expression of cartilage‐degrading proteases (MMP‐13, ADAMTS‐5 and MMP‐3) and chondrogenic markers (COL2A1, SOX‐9 and Aggrecan) in isolated human chondrocytes from Non‐OA (*n* = 20) and OA patients (*n* = 20) treated with either PBS (vehicle control) or rIL‐33 (30 ng mL^−1^, 24 h). **(c)** MMP‐13 and **(d)** MMP‐3 protein level in cell media supernatants obtained from isolated human chondrocytes from Non‐OA (*n* = 12) and OA patients (*n* = 12) treated with either PBS (vehicle control) or rIL‐33 (30 ng mL^−1^; 24 h). **(e, f)** OARSI scoring of cartilage tissue, **(g)** synovitis scoring and **(h)** osteophyte maturity scoring of sham‐operated (*n* = 20) or DMM‐operated (*n* = 20) WT mice (12 weeks post‐surgery end timepoint) treated intraperitoneally with either PBS (vehicle control) or rIL‐33 (33 μg kg^−1^; daily for 12 weeks post‐surgery). **(i)** von Frey pain assessment of sham‐operated (*n* = 20) or DMM‐operated (*n* = 20) WT mice (12 weeks post‐surgery end timepoint) treated intraperitoneally with either PBS (vehicle control) or rIL‐33 (33 μg kg^−1^; daily for 12 weeks post‐surgery). **(j)** mRNA expression of cartilage‐degrading proteases (MMP‐13, ADAMTS‐5 and MMP‐3) and chondrogenic markers (COL2A1, SOX‐9 and Aggrecan) in whole knee joints of DMM‐operated (*n* = 20) WT mice (12 weeks post‐surgery end timepoint) treated intraperitoneally with either PBS (vehicle control) or rIL‐33 (33 μg kg^−1^; daily for 12 weeks post‐surgery). All RT‐qPCR gene expressions were normalised to the endogenous level of 18 s in respective groups. Data are expressed as mean ± S.E.M. with unpaired 2‐tailed Student’s *t*‐tests (**c, d**), two‐way analysis of variance followed by the Tukey‐Kramer test (**f, g, h**), or repeated measures 2‐way ANOVA with Bonferroni’s post hoc tests was used to compare groups at each time point (i; DMM PBS vs DMM rIL‐33). *n* indicates the number of human specimens or mice per group. NS = non‐significant. *P* < 0.01, *P* < 0.001 or *P* < 0.0001 are represented as **, *** or ****, respectively.

We wanted to elucidate the outcome of elevating IL‐33 in human chondrocytes. Isolated chondrocytes treated with rIL‐33 displayed elevated mRNA and protein expression of cartilage‐degrading proteases (MMP‐13, MMP‐3 and ADAMTS‐5) and decreased levels of chondrogenic markers (COL2A1, SOX‐9 and Aggrecan) compared to cells treated with PBS (Figure 2a and b). Isolated chondrocytes treated with rIL‐33 also displayed increased release of MMP‐13 and MMP‐3 (Figure 2c and d). After these observations, we administered rIL‐33 in mice and then performed DMM surgery to confirm whether elevated levels of IL‐33 may be responsible for increased OA. Mice administered with rIL‐33 demonstrated increased cartilage degradation, synovitis, osteophyte maturity and pain compared to mice treated with PBS (Figure 2e–i and Supplementary figure 2a). In addition, mice administered with rIL‐33 had increased mRNA and protein levels of cartilage‐degrading proteases and lowered mRNA and protein levels of chondrogenic markers compared to mice treated with PBS (Figure 2j and Supplementary figure 2b, c); the same response as observed in human chondrocytes treated with rIL‐33.

### Synovial fibroblast‐specific ablation of IL‐33 decreases synovitis but does not affect cartilage degradation or pain

**Figure 3:**
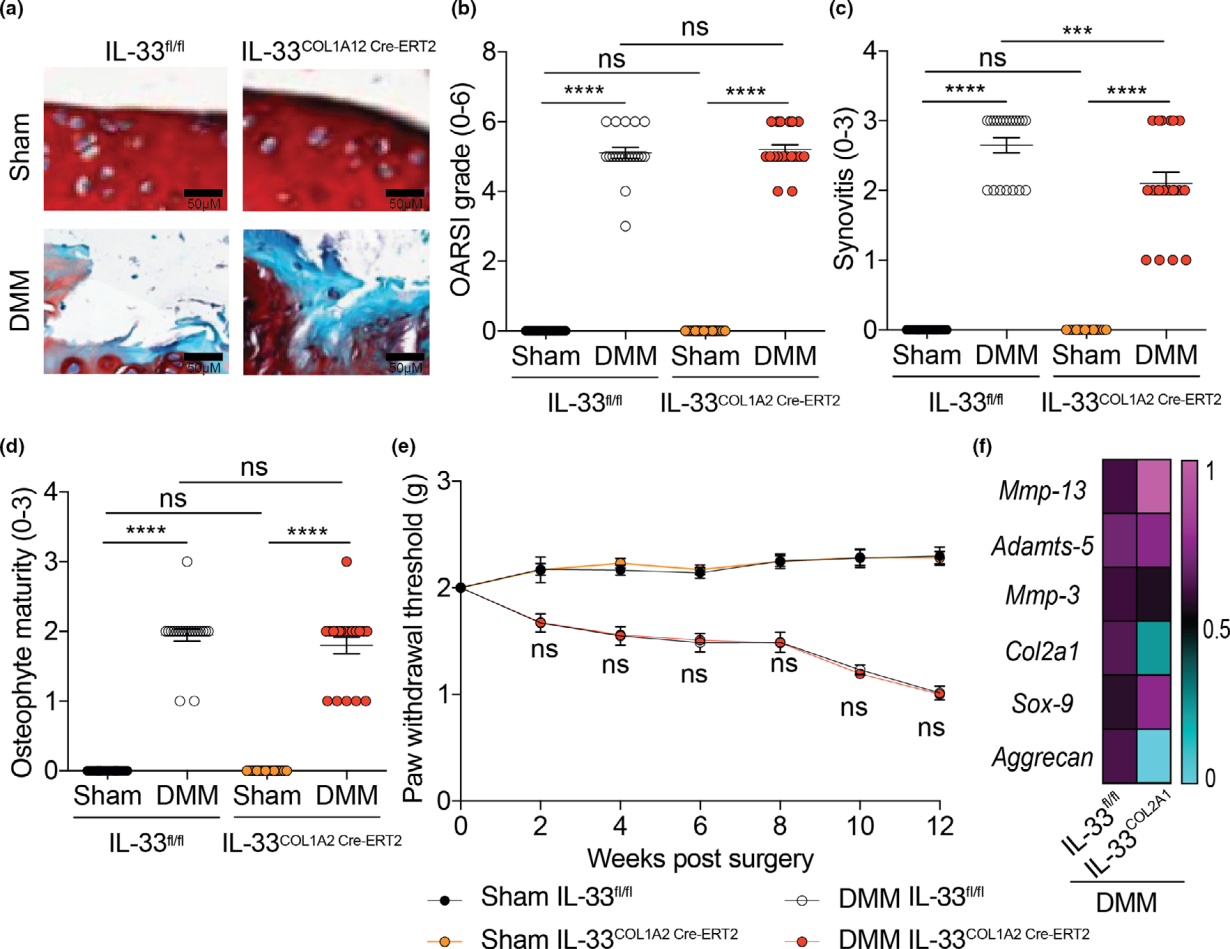
Synovial fibroblast‐specific ablation of IL‐33 decreases synovitis but does not affect cartilage degradation or pain. **(a, b)** OARSI scoring of cartilage tissue, **(c)** synovitis scoring and **(d)** osteophyte maturity scoring of sham‐operated (*n* = 20) or DMM‐operated (*n* = 20) IL‐33^fl/fl^ control mice and IL‐33^COL1A2 Cre‐ERT2^ mice (12 weeks post‐surgery end timepoint). **(e)** von Frey pain assessment of sham‐operated (*n* = 20) or DMM‐operated (*n* = 20) IL‐33^fl/fl^ control mice and IL‐33^COL1A2 Cre‐ERT2^ mice (12 weeks post‐surgery end timepoint). **(f)** mRNA expression of cartilage‐degrading proteases (MMP‐13, ADAMTS‐5 and MMP‐3) and chondrogenic markers (COL2A1, SOX‐9 and Aggrecan) in whole knee joints of DMM‐operated (*n* = 20) IL‐33^fl/fl^ control mice and IL‐33^COL1A2 Cre‐ERT2^ mice (12 weeks post‐surgery end timepoint). All RT‐qPCR gene expressions were normalised to the endogenous level of 18 s in respective groups. Data are expressed as mean ± S.E.M. with two‐way analysis of variance followed by the Tukey‐Kramer test **(b, c, d)** or repeated measures 2‐way ANOVA with Bonferroni’s post hoc tests was used to compare groups at each time point (e; DMM IL‐33^fl/fl^ control mice vs DMM IL‐33^COL1A2 Cre‐ERT2^ mice). *n* indicates the number of human specimens or mice per group. NS = non‐significant. *P* < 0.0001 is represented as ****.

Previous studies have showed IL‐33 to be produced by synovial fibroblasts in RA.^18^, ^19^ Hence, we wanted to investigate whether ablating IL‐33 in synovial fibroblasts will affect OA progression *in vivo*. We successful generated synovial fibroblast‐specific IL‐33 conditional KO mice (IL‐33^COL1A2 Cre‐ERT2^; Supplementary figure 3a, b) and induced OA in these mice using the DMM model. IL‐33^COL1A2 Cre‐ERT2^ mice displayed decreased synovitis, but there was no significant change in cartilage degradation or pain outcomes compared to IL‐33^fl/fl^ control mice (Figure 3a–e and Supplementary figure 3c). There was no significant difference in the expression of cartilage‐degrading proteases or chondrogenic markers in IL‐33^COL1A2 Cre‐ERT2^ mice compared to IL‐33^fl/fl^ control mice (Figure 3f and Supplementary figure 3d, e). Surprised by these results, we examined whether IL‐33 expression was changed in whole knee joints of IL‐33^COL1A2 Cre‐ERT2^ mice with OA. Whole knee joints with OA displayed no change in IL‐33 expression in IL‐33^COL1A2 Cre‐ERT2^ compared to IL‐33^fl/fl^ control mice (Supplementary figure 3f). This led us to hypothesise that the increased IL‐33 expression seen in OA joints may come from chondrocytes.

### Cartilage‐specific ablation of IL‐33 decreases cartilage degradation, synovitis and pain

**Figure 4:**
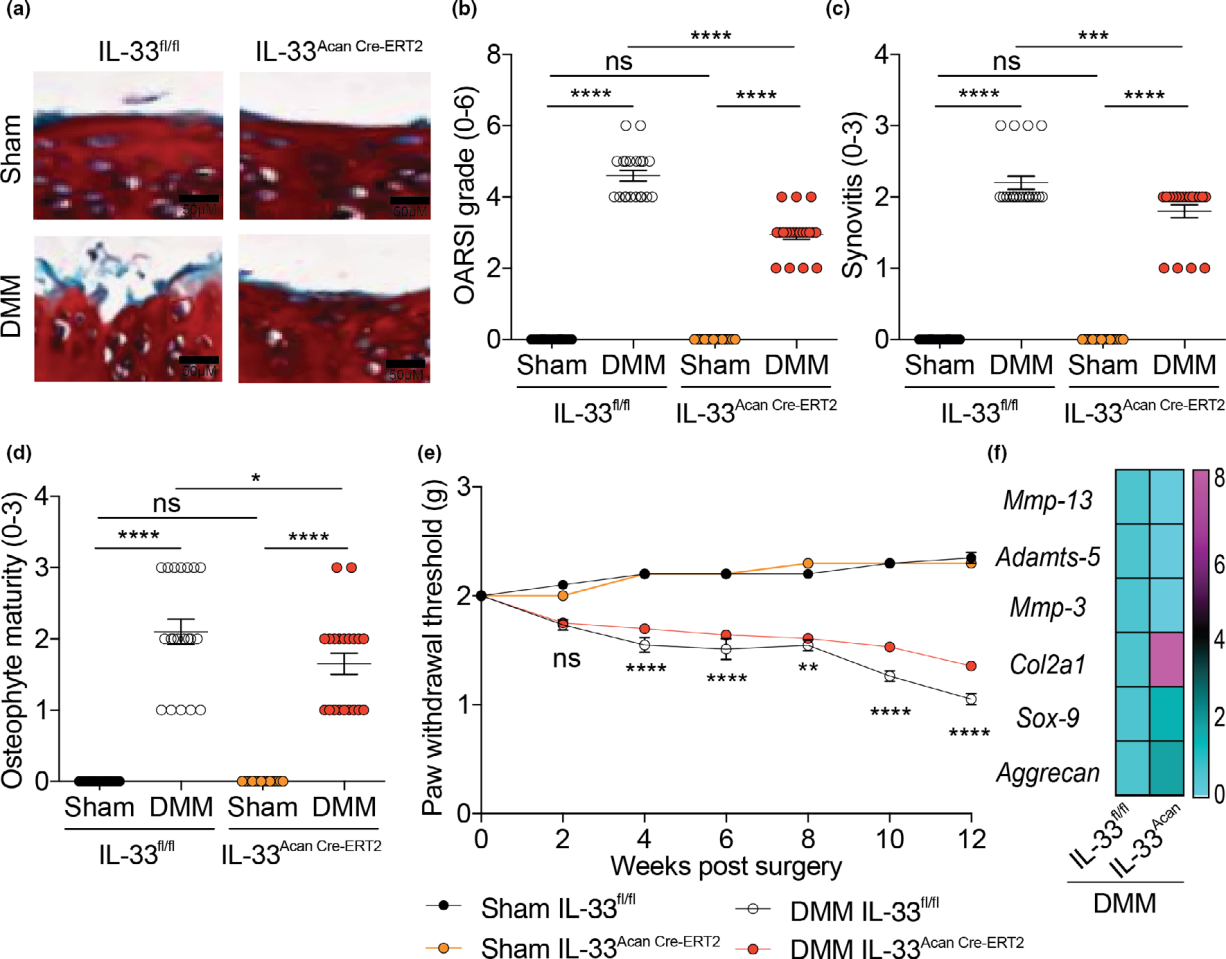
Cartilage‐specific ablation of IL‐33 decreases cartilage degradation, synovitis and pain. **(a, b)** OARSI scoring of cartilage tissue, **(c)** synovitis scoring and **(d)** osteophyte maturity scoring of sham‐operated (*n* = 20) or DMM‐operated (*n* = 20) IL‐33^fl/fl^ control mice and IL‐33^Acan Cre‐ERT2^ mice (12 weeks post‐surgery end timepoint). **(e)** von Frey pain assessment of sham‐operated (*n* = 20) or DMM‐operated (*n* = 20) IL‐33^fl/fl^ control mice and IL‐33^Acan Cre‐ERT2^ mice (12 weeks post‐surgery end timepoint). **(f)** mRNA expression of cartilage‐degrading proteases (MMP‐13, ADAMTS‐5 and MMP‐3) and chondrogenic markers (COL2A1, SOX‐9 and Aggrecan) in whole knee joints of DMM‐operated (*n* = 20) IL‐33^fl/fl^ control mice and IL‐33^Acan Cre‐ERT2^ mice (12 weeks post‐surgery end timepoint). All RT‐qPCR gene expressions were normalised to the endogenous level of 18 s in respective groups. Data are expressed as mean ± S.E.M. with two‐way analysis of variance followed by the Tukey‐Kramer test (**b, c, d**) or repeated measures 2‐way ANOVA with Bonferroni’s post hoc tests was used to compare groups at each time point (e; DMM IL‐33^fl/fl^ control mice vs DMM IL‐33^Acan Cre‐ERT2^ mice). *n* indicates the number of human specimens or mice per group. NS = non‐significant. *P* < 0.05, *P* < 0.001 or *P* < 0.0001 are represented as *, *** or ****, respectively.

We next successfully generated cartilage‐specific IL‐33 conditional KO mice (IL‐33^Acan Cre‐ERT2^; Supplementary figure 4a, b) to investigate whether IL‐33 production from chondrocytes is the main factor in driving OA. IL‐33^Acan Cre‐ERT2^ mice displayed decreased cartilage degradation, synovitis and pain compared to IL‐33^fl/fl^ control mice post‐DMM surgery (Figure 4a–e and Supplementary figure 4c). In addition, whole knee joints of IL‐33^Acan Cre‐ERT2^ mice displayed decreased expression of cartilage degrading proteases and increased expression of chondrogenic markers in IL‐33^Acan Cre‐ERT2^ mice compared to IL‐33^fl/fl^ control mice post‐DMM surgery (Figure 4f and Supplementary figure 4d, e). We also observed a significant reduction of IL‐33 mRNA expression in whole knee joints of IL‐33^Acan Cre‐ERT2^ mice compared to IL‐33^fl/fl^ control mice post‐DMM surgery (Supplementary figure 4f), indicating IL‐33 from chondrocytes mainly drives disease progression *in vivo*.

### Neutralising ST2 and IL‐33 attenuates OA

**Figure 5:**
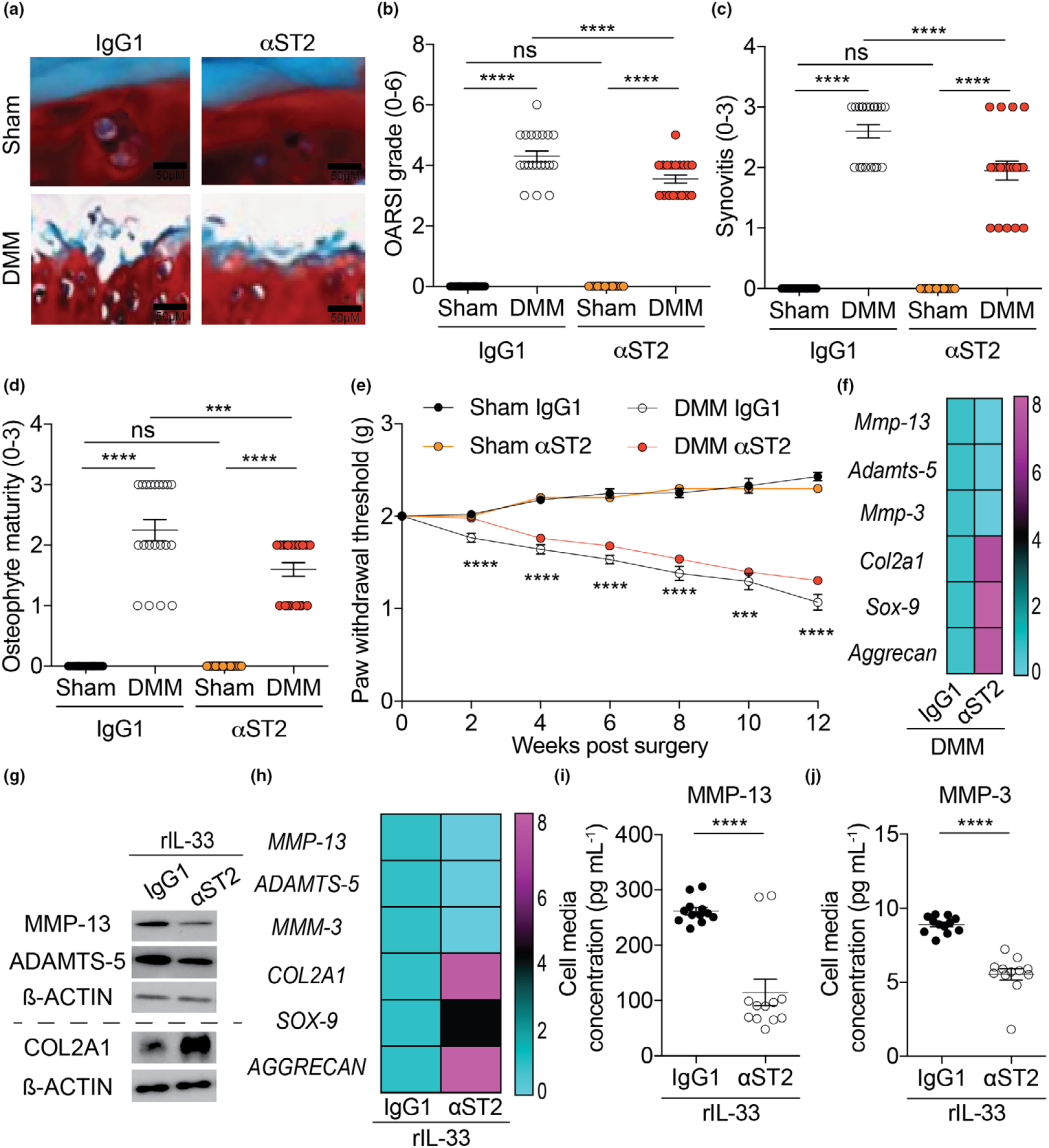
Neutralising ST2 attenuates OA. **(a, b)** OARSI scoring of cartilage tissue, **(c)** synovitis scoring and **(d)** osteophyte maturity scoring of sham‐operated (*n* = 20) or DMM‐operated (*n* = 20) WT mice (12 weeks post‐surgery end timepoint) treated intraperitoneally with either IgG1 (vehicle control; 50 µg per mouse; daily for 12 weeks post‐surgery) or αST2 (50 µg per mouse; daily for 12 weeks post‐surgery). **(e)** von Frey pain assessment of sham‐ (*n* = 20) or DMM‐ (*n* = 20) operated WT mice (12 weeks post‐surgery end timepoint) treated intraperitoneally with either IgG1 (vehicle control; 50 µg per mouse; daily for 12 weeks post‐surgery) or αST2 (50 µg per mouse; daily for 12 weeks post‐surgery). **(f)** mRNA expression of cartilage‐degrading proteases (MMP‐13, ADAMTS‐5 and MMP‐3) and chondrogenic markers (COL2A1, SOX‐9 and Aggrecan) in whole knee joints of DMM‐operated (*n* = 20) treated intraperitoneally with either IgG1 (vehicle control; 50 µg per mouse; daily for 12 weeks post‐surgery) or αST2 (50 µg per mouse; daily for 12 weeks post‐surgery). **(g)** protein and **(h)** mRNA expression of cartilage degrading proteases (MMP‐13, ADAMTS‐5 and MMP‐3) and chondrogenic markers (COL2A1, SOX‐9 and Aggrecan) in isolated human chondrocytes from Non‐OA (*n* = 20) and OA patients (*n* = 20) treated with rIL‐33 (30 ng mL^−1^, 24 h) and IgG1 (vehicle control; 3 μg mL^−1^, 24 h) or αST2 (3 μg mL^−1^, 24 h). **(i)** MMP‐13 **(j)** MMP‐3 protein level in cell media supernatants obtained from isolated human chondrocytes from Non‐OA (*n* = 12) and OA patients (*n* = 12) treated with rIL‐33 (30 ng mL^−1^; 24 h) and IgG1 (vehicle control; 3 μg mL^−1^, 24 h) or αST2 (3 μg mL^−1^, 24 h). All RT‐qPCR gene expressions were normalised to the endogenous level of 18 s in respective groups. Data are expressed as mean ± S.E.M. with two‐way analysis of variance followed by the Tukey‐Kramer test **(b, c, d)** or repeated measures 2‐way ANOVA with Bonferroni’s post hoc tests was used to compare groups at each time point (**e**; DMM IgG1 control mice vs DMM αST2 mice) or with unpaired 2‐tailed Student’s *t*‐tests **(i, j)**. *n* indicates the number of human specimens or mice per group. NS = non‐significant. *P* < 0.001 or *P* < 0.0001 are represented as *** or ****, respectively.

We wanted to investigate whether blocking IL‐33 signalling may attenuate OA *in vivo*. WT mice treated with ST2‐neutralising antibody demonstrated decreased cartilage degradation, synovitis and pain compared to IgG1‐treated control mice post‐DMM surgery (Figure 5a–e and Supplementary figure 5a). Neutralising ST2 reduced expression of cartilage degrading proteases and increased expression of chondrogenic markers in mice post‐DMM surgery (Figure 5f and Supplementary figure 5b, c) and in isolated OA human chondrocytes treated with rIL‐33 (Figure 5g and h) compared to respective controls. In addition, the increased release of MMP‐13 and MMP‐3 induced by rIL‐33 was reduced with the treatment of αST2 in isolated OA human chondrocytes compared to IgG1‐treated human OA chondrocytes (Figure 5i and j).

**Figure 6:**
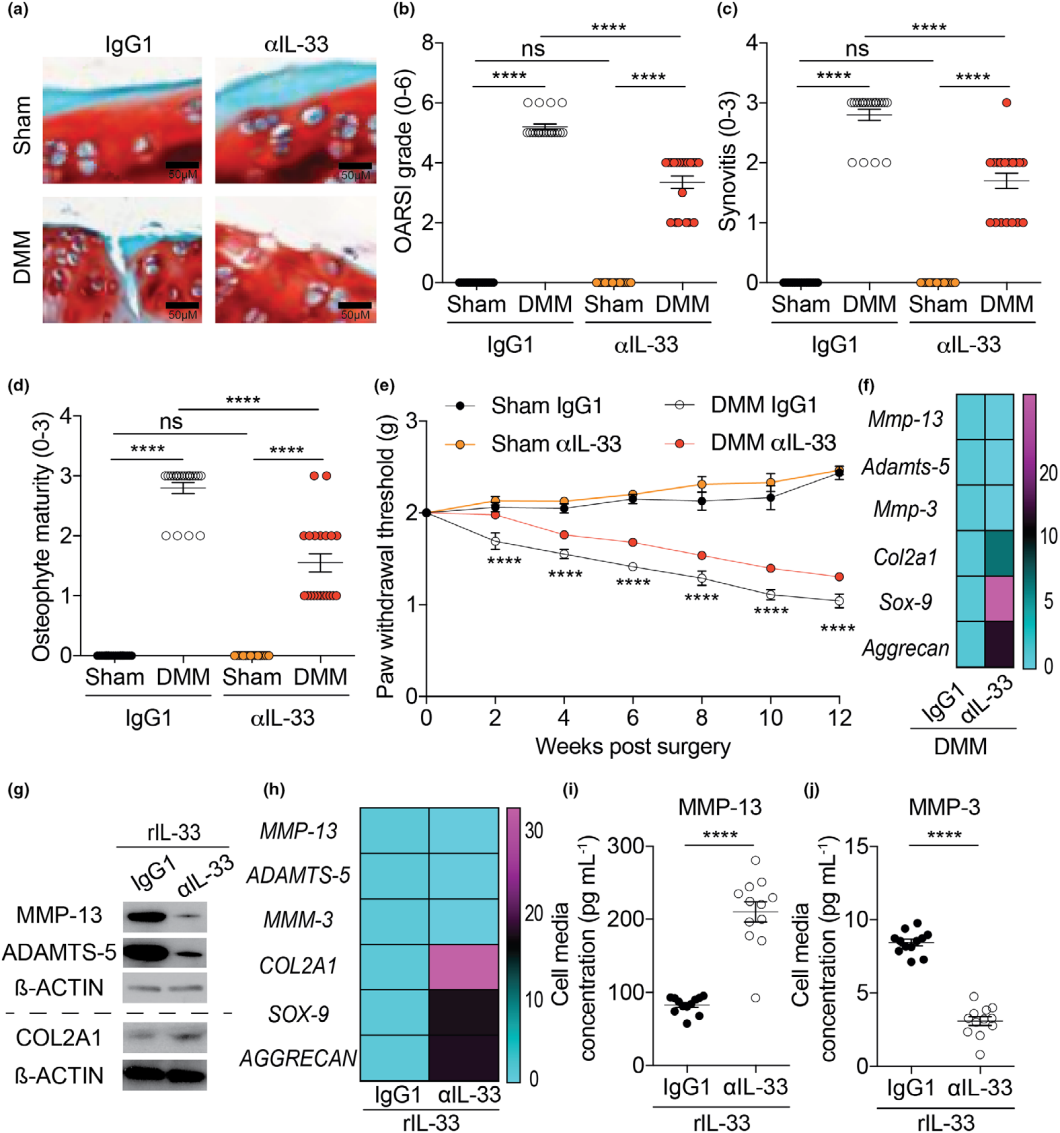
Neutralising IL‐33 attenuates OA. **(a, b)** OARSI scoring of cartilage tissue, **(c)** synovitis scoring and **(d)** osteophyte maturity scoring of sham‐operated (*n* = 20) or DMM‐operated (*n* = 20) WT mice (12 weeks post‐surgery end timepoint) treated intraperitoneally with either IgG1 (vehicle control; 15 µg per mouse; daily for 12 weeks post‐surgery) or αIL‐33 (15 µg per mouse; daily for 12 weeks post‐surgery). **(e)** von Frey pain assessment of sham‐operated (*n* = 20) or DMM‐operated (*n* = 20) WT mice (12 weeks post‐surgery end timepoint) treated intraperitoneally with either IgG1 (vehicle control; 15 µg per mouse; daily for 12 weeks post‐surgery) or αIL‐33 (15 µg per mouse; daily for 12 weeks post‐surgery). **(f)** mRNA expression of cartilage degrading proteases (MMP‐13, ADAMTS‐5 and MMP‐3) and chondrogenic markers (COL2A1, SOX‐9 and Aggrecan) in whole knee joints of DMM‐operated (*n* = 20) treated intraperitoneally with either IgG1 (vehicle control; 15 µg per mouse; daily for 12 weeks post‐surgery) or αIL‐33 (15 µg per mouse; daily for 12 weeks post‐surgery). **(g)** protein and **(h)** mRNA expression of cartilage degrading proteases (MMP‐13, ADAMTS‐5 and MMP‐3) and chondrogenic markers (COL2A1, SOX‐9 and Aggrecan) in isolated human chondrocytes from Non‐OA (*n* = 20) and OA patients (*n* = 20) treated with rIL‐33 (30 ng mL^−1^, 24 h) and IgG1 (vehicle control; 10 μg mL^−1^, 24 h) or αIL‐33 (10 μg mL^−1^, 24 h). **(i)** MMP‐13 (**j)** MMP‐3 protein level in cell media supernatants obtained from isolated human chondrocytes from Non‐OA (*n* = 12) and OA patients (*n* = 12) treated with rIL‐33 (30 ng mL^−1^; 24 h) and IgG1 (vehicle control; 10 μg mL^−1^, 24 h) or αIL‐33 (10 μg mL^−1^, 24 h). All RT‐qPCR gene expressions were normalised to the endogenous level of 18 s in respective groups. Data are expressed as mean ± S.E.M. with two‐way analysis of variance followed by the Tukey‐Kramer test (**b, c, d**) or repeated measures 2‐way ANOVA with Bonferroni’s post hoc tests was used to compare groups at each time point (**e**; DMM IgG1 control mice vs DMM αIL‐33 mice) or with unpaired 2‐tailed Student’s *t*‐tests (**i, j**). *n* indicates the number of human specimens or mice per group. NS = non‐significant. *P* < 0.0001 is represented as ****.

Finally, we used an IL‐33‐neutralising antibody in mice post‐DMM surgery. WT mice treated with αIL‐33 displayed decreased cartilage degradation, synovitis and pain compared to IgG1‐treated control mice post‐DMM surgery (Figure 6a–e and Supplementary figure 6a). αIL‐33 treatment also reduced expression of cartilage‐degrading proteases and increased expression of chondrogenic markers in mice (Figure 6f and Supplementary figure 6b, c) and isolated OA human chondrocytes (Figure 6g and h) compared to respective controls. The increased release of MMP‐13 and MMP‐3 induced by rIL‐33 in human OA chondrocytes was also reduced by αIL‐33 treatment (Figure 6i and j).

## Discussion

We have shown that IL‐33 and ST2 are elevated in human and murine OA. Increasing levels of IL‐33 exacerbates disease in mice and in turn increases the expression of cartilage degrading proteases (MMP‐13, MMP‐3 and ADAMTS‐5) and reduces chondrogenic markers (COL2A1, SOX‐9 and Aggrecan). Interestingly, we also observed IL‐33 production in an OA joint is mainly from chondrocytes and not synovial fibroblasts. Most importantly, we report that neutralising IL‐33 and ST2 reduces cartilage degradation and pain *in vivo*.

Throughout our experiments, we observed increasing IL‐33 (via rIL‐33) in turn increased the expression and production of cartilage‐degrading proteases and reduced the expression of chondrogenic markers in both human and murine models. Vice versa decreasing IL‐33 signalling in turn reduced the expression and production of cartilage‐degrading proteases and increased the expression of prochondrogenic markers in both human and murine models. These strong correlations suggest IL‐33 signalling acts in the same manner as IL‐1 and TNF‐α signalling to regulate cartilage proteases, aggrecanases and matrix synthesis.^3^, ^24^ We did not investigate the mechanistic pathway of how IL‐33 regulates these genes and proteins in chondrocytes. However, Li and colleagues have demonstrated double‐stranded RNA released from damaged articular chondrocytes increased IL‐33 expression via the TLR3/p38 pathway to increase MMP‐13 and MMP‐3 production and inhibit COL2A1 expression.^25^

The cell type responsible for IL‐33 production in an OA joint was of interest to us after observing our initial findings of increased IL‐33 expression in OA patients. Palmer and colleagues showed IL‐33 from synovial fibroblasts was important in RA pathogenesis.^20^ Hence, we were surprised to observe that synovial fibroblast‐specific IL‐33 KO mice did not display significantly reduced IL‐33 gene expression in whole knee joints post‐DMM surgery or have reduced disease outcomes compared to control mice. Having observed this result, we sought to investigate whether chondrocytes were responsible for IL‐33 production in an OA joint. Chondrocyte‐specific IL‐33‐deficient mice did display better disease outcomes compared to control mice post‐DMM surgery. Moreover, IL‐33 production in whole knee joints of these mice was significantly reduced compared to control mice. Together, these results demonstrate that chondrocytes are responsible for IL‐33 production in an OA joint and the immune response of IL‐33 may be different in RA and OA. This may suggest why the use of anti‐TNFα drugs in OA patients has not been successful compared to the results seen in RA patients.^26^

We also noticed synovial fibroblast‐specific IL‐33 KO mice showed reduced synovitis, but this did not impact cartilage degradation, protease and chondrogenic marker expression and pain post‐DMM surgery. This result raises an interestingly larger question if the synovitis seen in an OA joint driven by cytokines and cells in the synovium are important in OA pathogenesis compared to the cytokine production by chondrocytes. However, further investigations on the crosstalk between IL‐33 and other cytokines and cell types in an OA joint have to be conducted to answer this larger question. It must be noted the COL1A2 Cre‐ERT2 is not only synovial fibroblast specific but can also be specific to dermal and visceral fibroblasts.^27^ However, we did not note any adverse effects in these tissues. Currently, this is the only viable Cre‐ERT2 available to target synovium fibroblast cells; hence, we used this murine tool in our experiments.

The most important translational finding of our study was that neutralising IL‐33 and ST2 improved OA disease outcomes *in vivo*. IL‐1 and TNF‐α have been extensively shown to be present in an OA joint and to drive disease pathogenesis.^2^, ^3^ Past studies have tried to target pro‐inflammatory cytokines such as IL‐1 and TNF‐α and their respective receptors for OA therapy but showed poor efficacy. The shared common cellular and intercellular pathways between IL‐33 and IL‐1 and TNF‐α may suggest targeting IL‐33 signalling is a possible new and better approach to address OA. It must be noted we only used systemic administration of both neutralising antibodies because of the required daily dosing regimen. Daily intra‐articular injections of both neutralising antibodies over a 12‐week period in itself caused joint damage hence were not plausible to conduct in these studies. This may suggest these drugs and reagents may have a whole‐body immune modulation, which may have subsequent effect in the joint. In addition, we only used the DMM model of OA. Using other models such as the anterior cruciate ligament transection model may further substantiate our findings. A further limitation of our study was not using a spontaneous ageing model of OA. IL‐33 signalling has been shown to be involved in the exacerbation of age‐related diseases.^7^ In speculation, our data suggest it is plausible for IL‐33 signalling to be detrimental to cartilage homeostasis during ageing. In addition, we could not access osteophyte formation and bone changes during disease development in mice using micro‐CT analysis. Hence, the role of IL‐33 and ST2 in an aged joint and other joint tissues still need to be further investigated.

To our knowledge, this is the first time neutralising IL‐33 and ST2 has been demonstrated to reduce OA and pain *in vivo*. These results show that IL‐33 and ST2 blockade may also be an effective analgesic for OA. A study by Zarpelon and colleagues demonstrated carrageenin‐induced paw oedema and hyperalgesia were reduced in ST2 KO mice.^28^ Further studies on the relationship between IL‐33 and local and systemic pain signalling networks in an OA joint should be conducted.

**Figure 7:**
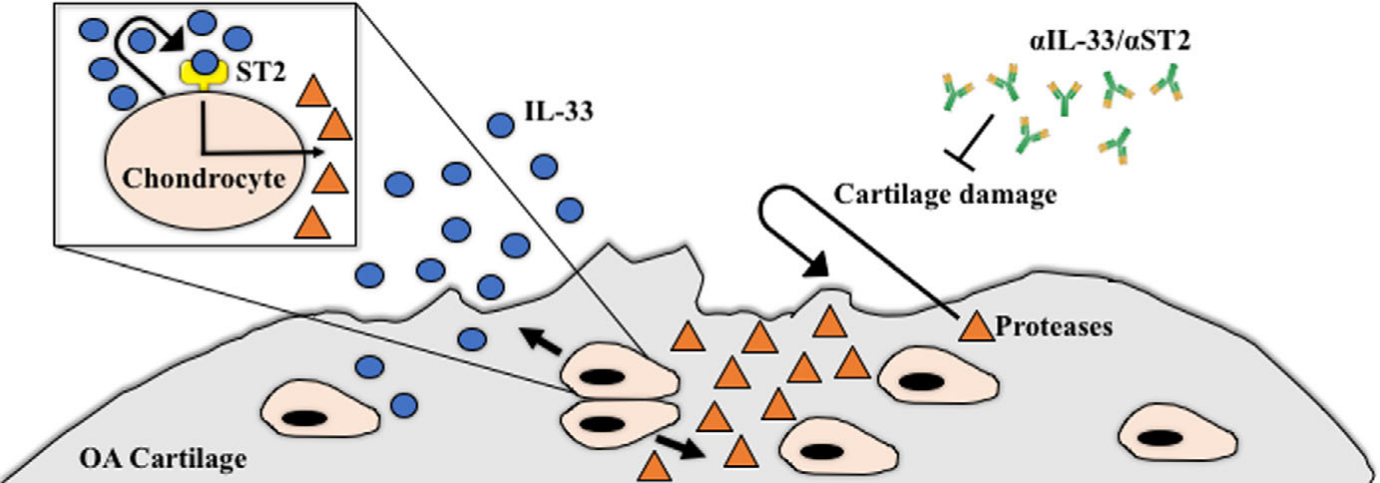
Blockade of IL‐33 signalling attenuates OA. IL‐33 is produced locally in OA joints by chondrocytes. Neutralising IL‐33 and ST2 antibodies attenuates the severity of OA and pain *in vivo* and decreases the production of cartilage degrading proteases.

In conclusion, we report that IL‐33 and its receptor ST2 are expressed in human and mouse OA chondrocytes. Our findings indicate that IL‐33 is produced locally in OA joints by chondrocytes and not synovial fibroblasts. Administration of neutralising IL‐33 and ST2 antibodies attenuated the severity of OA and pain *in vivo* and was associated with a marked decrease in the production of cartilage‐degrading proteases alongside an increase expression of chondrogenic markers. Neutralisation of IL‐33 signalling may prove to be a therapeutic option for OA (Figure 7).

## Methods

### Human samples

Healthy or OA knee/hip cartilage, SF and serum were obtained from OA patients at The Second Hospital of Nanjing. OA patients were a mix of both females and males (age range from 42 to 79) and were either undergoing either hip or knee replacement surgery. Non‐OA samples were obtained from patients who had undergone surgery for tumors of the lower leg in which the knee joint was not affected males (age range from 39 to 76). Tissue samples were collected with informed written consent in full compliance with Declaration of Helsinki and approval of the Ethics Committee of The Second Hospital of Nanjing. All samples were transferred to the laboratory within 2 h. Dissected cartilage pieces were incubated overnight in Dulbecco's modified Eagle's medium (DMEM; Lonza, Basel) with 1 mg mL^−1^ Collagenase A (Roche Pharmaceuticals, Basel) at 37°C for 5–6 h to isolate chondrocytes. Cells used in experiments were at passage 1. Serum was obtained from whole blood by centrifugation at 1600 *g* for 15 min at 20°C. Synovial fluid (SF) was centrifuged for 20 min at 3000 *g*. Serum and SF were stored at −80°C before experimentation.

### Cell culture

Isolated human chondrocytes were cultured in DMEM media containing 4.5 g L^−1^ of glucose and L‐glutamine (Lonza). DMEM was supplemented, unless stated otherwise with 10% foetal calf serum (FCS; Lonza), 1% Penicillin and Streptomycin (Lonza), Amphotericin B (Gibco, Waltham) and 2% (4‐(2‐hydroxyethyl)‐1‐piperazineethanesulfonic acid solution (Lonza). Cells were maintained under sterile conditions in a humidified atmosphere of 37°C containing 95% oxygen and 5% (v/v) carbon dioxide unless stated otherwise.

### *In vitro* treatments

Isolated human chondrocytes were cultured to confluence, serum starved with 1% FCS DMEM media for 1 h and then treated with either PBS (Thermo Fisher Scientific, Waltham) vehicle control or human recombinant (r)IL‐33 (30 ng mL^−1^; PeproTech) for 24 h. For some experiments, IgG1 isotype control (3 μg mL^−1^ or 10 μg mL^−1^; Sigma‐Aldrich, St. Louis) or human neutralising antibody (α)ST2 (3 μg mL^−1^; R&D Systems, Minneapolis) or αIL‐33 (10 μg mL^−1^; R&D Systems) was added alongside human rIL‐33 (30 ng mL^−1^) for 24 h.

### RNA analysis of human cells

Total RNA was isolated from human cell cultures using RNeasy mini kit (Qiagen, Hilden) and reverse transcribed using a High Capacity reverse transcription cDNA kit (Applied Biosystems, Foster City) according to the recommendations of the manufacturer. RT‐qPCR was carried out on a Real‐Time PCR System (Zeesan systems, Xiamen). Relative gene expression was analysed by the ∆∆Ct method using 18S as an endogenous control gene and TaqMan probes (Supplementary table 1).

### Mice

All animal procedures were approved by local ethics committee. Male mice weighed 18–25 g and were maintained in cages under controlled temperatures (19–23°C) and lights (12‐h light, 12‐h dark cycle). All mice had *ad libitum* access to water and chow. C57BL/6J (Charles River, Wilmington) wild‐type (WT) mice were used unless otherwise stated.

### Generation of synovial fibroblast‐ and cartilage‐specific IL‐33 conditional KO mice

Male mice carrying a floxed IL‐33 allele (IL‐33^fl/fl^; Jackson Laboratory, Bar Harbor) were bred with homozygote mice carrying transgenes for COL1A2 Cre‐ERT2 (Jackson Laboratory) or Aggrecan Cre‐ERT2 (Jackson Laboratory). Hence, we generated two different types of homozygote double transgenic tissue‐specific conditional knockout (KO) mice. (1) IL‐33^fl/fl^; COL1A2 Cre‐ERT2 (IL‐33^COL1A2 Cre‐ERT2^) that are synovial‐specific IL‐33‐conditional KO mice. (2) Aggrecan Cre‐ERT2 (IL‐33^Acan Cre‐ERT2^) that are cartilage‐specific IL‐33‐conditional KO mice. Mice were obtained at the expected Mendelian ratio with no adverse phenotypic side effects. Adult mice (8 weeks of age) were administered intraperitoneal doses of free base tamoxifen (TX) (2 mg kg^−1^; Sigma‐Aldrich) three times every other day for one week to induce conditional KO of IL‐33. IL‐33^fl/fl^ mice were used as controls for conditional KO mice unless otherwise stated.

### Experimental OA

Murine OA was induced by using the destabilisation of the medial meniscus (DMM) model.^29^ 10‐week‐old male mice were anaesthetised. The medial meniscus was identified, and the attachments of its anterior horn to the tibial plateau were cut. For sham control limbs, the mice were anaesthetised and prepared as before. The right knee was opened using the same medial para‐patellar approach and the meniscus identified, but the menisco‐tibial ligament was not released. The incision was closed, and the mice recovered as before. Left (contralateral) knees for both sham and DMM operations were left as un‐operated controls.

### Pain assessment

Pain associated with OA was measured in mice using the von Frey test and the hot plate assay as previously described.^30^ Pain tests were conducted three times before sham or DMM surgery and then once every two weeks after surgery. For the hot plate assay, a surface of 55 ± 0.5°C was used with a latency period (cut‐off) of 30 s defined as complete analgesia. For the von Frey test, mice were allowed to acclimate for at least 15 min before mechanical allodynia was tested by touching the plantar surface of the hind paw with von Frey filaments in ascending order of force for up to 6 s.

### *In vivo* treatments

For experiments which increased IL‐33 levels in *vivo*, mouse rIL‐33 (Enzo Life Sciences, Farmingdale) was administered intraperitoneally (i.p) on a daily basis at 33 μg kg^−1^ for 12 weeks post‐surgery. PBS was used as vehicle control in experiments. For blocking IL‐33 and ST2 signalling, mouse αIL‐33 (15 µg per mouse) or mouse αST2 (50 µg per mouse) antibodies (R&D Systems) were dissolved in sterile PBS and injected in respective groups of mice daily via i.p. Isotype control IgG1 (Sigma‐Aldrich) was dissolved in sterile PBS and injected daily via i.p in mice at respective doses of each neutralising antibody.

### Extraction of murine tissues post‐DMM surgery

Whole blood was extracted from mice at 12 weeks post‐surgery left to clot at room temperature for 15 min, then centrifuged at 3000 g for 10 min to obtain serum. SF was extracted from culled mice (12 weeks post‐surgery) by injecting sterile PBS into the knee joint, then extracting the fluid. Knee joints were harvested by excising at the proximal femur and distal tibia. The skin and surrounding muscles were then removed without disturbing the joint and its associated ligaments. Knee joints were fixed in 10% neutral buffered formalin for at least 24 h before any further processing.

### Histology

The specimens were decalcified in 20% formic acid for 14 days. The knees were then processed as per standard protocol: 2 changes of formalin (40°C) followed by 70% alcohol (40°C), 90% alcohol (40°C), 3 changes of absolute alcohol (40°C), 4 changes of xylene (40°C) and 4 changes of paraffin wax (60°C) with each change lasting 1.5 h. The knees were then embedded in paraffin wax. 4‐μm‐thick coronal sections were cut and stained with Safranin‐O.

### Disease scoring

Cartilage destruction was scored by two blinded observers using the OARSI grading system. Briefly,^31^ a minimum of 6 sections were scored per joint. All four quadrants of the knee were individually scored (medial femoral condyle and tibial plateau, lateral femoral condyle and tibial plateau). The summed score was calculated from the sum of the three highest scoring section totals (obtained from the sum of all four quadrants per section). This provides an indication of both severity and extent of OA damage within the joint. There was no significant difference in the scores between the two blinded scorers. Synovitis was determined by grading synovial inflammation (grade 0–3) as previously described.^32^ Osteophyte maturity (grade 0–3) was quantified as previously described.^33^

### Extraction of murine cartilage protein

Hips from young mice at 7 weeks of age are cartilaginous and have adequate protein when pooled together (4 hips =  1*n*). These pooled hips were used to validate efficient knockdown of IL‐33 protein in conditional cartilage‐specific KO mice. Mice were injected with TX at 5 weeks of age and culled at week 7. Hips were avulsed and placed in ice‐cold protein lysis buffer (Sigma‐Aldrich; see Western blot analysis below). Thereafter, samples were shaken for 2 h at 4°C. The samples were then centrifuged at 13 000 × *g* for 5 min at 4°C, and the supernatants were collected for experiments. Immunoblotting was performed on the collected supernatants as described below.

### RNA analysis of murine tissues

Validation of efficient knockdown of IL‐33 mRNA expression in conditional cartilage specific KO mice was performed on microdissected cartilage pooled from 4 mouse knee joints (1*n*). Briefly, mice were injected with TX at 8 weeks of age and culled at week 10. Cartilage was microdissected and stored in RNAlater® stabilisation solution (Life technologies, Carlsbad) at −80°C, whereas whole knee joints for RNA analysis were exercised as stated above then stored for RNAlater® stabilisation solution (Life technologies) at −80°C for experimentation.

Samples were placed in TRIzol® (Life technologies), were homogenised, shaken for 10 min at room temperature and then centrifuged at 13 000 × *g* for 10 min at 4°C. Supernatants were diluted in an equal amount of 1‐Bromo‐3‐chloropropane (Sigma‐Aldrich), vortexed and centrifuged 13 000 × *g* for 15 min at 4°C. The upper aqueous phase was removed and placed in new Eppendorfs. Thereafter, the RNeasy microkit (Qiagen) protocol was followed for microdissected cartilage and the RNeasy mini kit (Qiagen) according to the manufacturer’s guidelines. RT‐qPCR was carried out as mentioned above.

### Extraction of synovial fibroblasts and culture

Murine synovial fibroblast isolation and culture was adapted from previously published protocol.^34^ Briefly, the articular cavity murine knee joints were cut open along both sides of the patella under a microscope to isolate the intra‑articular synovium carefully. Thereafter, the connective tissues around the synovium were carefully eliminated under a microscope. The synovium was finely chopped in DMEM media and 0.5 mL 1% type IV collagenase (Thermo Fisher Scientific) and incubated at a constant temperature of 37˚C in an orbital shaker incubator (200 rpm) for 60 min. Samples were vortexed vigorously for 1.5 min to release the cells. The samples were centrifuged for 5 min at 3000 × *g* and resuspended with DMEM supplemented with 10% FCS and 1% penicillin‑streptomycin. The cells were seeded and cultured (37˚C, 5% CO2) until confluence for RNA and protein extraction. Intra‑articular synovium of 10 murine knee joints had to be pooled together for 1*n*.

### Western blot analysis

Tissue or cell samples were homogenised in lysis buffer (RIPA buffer) (Sigma‐Aldrich), Ethylenediaminetetraacetic acid (EDTA)‐free protease inhibitor (Roche Pharmaceuticals), phosphatase inhibitor cocktail 2 and 3 (Sigma‐Aldrich) and protein levels quantified by bicinchoninic acid (BCA) assay (Thermo Fisher Scientific). Samples were probed overnight with primary antibody: IL‐33 (~ 30 kDa; 1:100; Novus Biologicals, Littleton; NBP1‐75516), ST2 (~ 50 and ~ 60 kDa; 1:100; Novus Biologicals; NBP2‐53096), MMP‐13 (~ 60 kDa; 1:100; Abcam, Cambridge; ab39012), ADAMTS‐5 (~ 73 kDa; 1:100; Abcam; ab41037), COL2A1 (~ 141 kDa 1:1000; Sigma‐Aldrich; SAB4500366) and β‐actin (loading control) (~ 42 kDa; 1:20 000; Sigma‐Aldrich; A2228).

### Immunohistochemistry

Heat‐mediated antigen retrieval was performed on paraffin‐embedded sections using citric acid buffer (Sigma‐Aldrich) warmed in a water bath at 100°C for 20 min. MMP‐13 (Abcam) antibody, COL2A1 (Sigma‐Aldrich) antibody and rabbit IgG control antibody (Sigma‐Aldrich) were used at 1:200 dilution. Quantification of positive cells/total cells was counted by two blinded operators.

### ELISA

Stored supernatants or SF were defrosted at room temperature, and MMP‐13 and MMP‐3 concentrations were measured via ELISA (R&D Systems). Assays were conducted as per the manufacturer’s instructions. Each ELISA sample was measured twice, and the average was used to plot the data (CV < 9%). ELISA experiments were conducted by blinded experimenters.

### Data analysis

All data are expressed as mean ± standard error of mean (S.E.M.) of *n* observations. Experiments were statistically analysed utilising the Student’s unpaired two‐tailed *t*‐test or analysed with two‐way analysis of variance followed by the Tukey‐Kramer test or repeated measures 2‐way ANOVA with Bonferroni’s post hoc tests. A significant difference was accepted when *P* < 0.05, *P* < 0.01, *P* < 0.001 or *P* < 0.0001 represented in all tables and figures as *, **, *** or ****, respectively. Data analysis was performed using GraphPad Prism^®^ 5.0 (GraphPad Software, California).

## Conflict of interest

The authors declare no conflict of interest.

## Author Contributions

**Zengliang He:** Data curation; Formal analysis; Investigation; Methodology; Project administration; Writing‐review & editing. **Yan Song:** Data curation; Formal analysis; Investigation; Methodology. **Yongxiang Yi:** Data curation; Funding acquisition; Methodology; Project administration; Supervision. **Fenzuo Qiu:** Data curation; Methodology; Validation. **Junhua Wang:** Data curation; Methodology; Validation. **Qingwen Jin:** Data curation; Funding acquisition; Investigation; Methodology; Supervision; Writing‐review & editing. **Junwei Li:** Funding acquisition; Investigation; Methodology; Supervision; Writing‐review & editing. **Pradeep Kumar Sacitharan:** Conceptualization; Data curation; Formal analysis; Funding acquisition; Investigation; Methodology; Project administration; Supervision; Validation; Visualization; Writing‐original draft; Writing‐review & editing.

## Data Availability

The data that support the findings of this study are available from the corresponding author upon reasonable request.

## References

[1] Glyn‐Jones S , Palmer AJ , Agricola R *et al* Osteoarthritis. Lancet 2015; 386: 376–387. 25748615 10.1016/S0140-6736(14)60802-3

[2] Kapoor M , Martel‐Pelletier J , Lajeunesse D , Pelletier JP , Fahmi H . Role of proinflammatory cytokines in the pathophysiology of osteoarthritis. Nat Rev Rheumatol 2011; 7: 33–42. 21119608 10.1038/nrrheum.2010.196

[3] Goldring MB , Otero M . Inflammation in osteoarthritis. Curr Opin Rheumatol 2011; 23: 471–478. 21788902 10.1097/BOR.0b013e328349c2b1 PMC3937875

[4] Sacitharan PK , Snelling SJ , Edwards JR . Aging mechanisms in arthritic disease. Discov Med 2012; 14: 345–352. 23200066

[5] Calich AL , Domiciano DS , Fuller R . Osteoarthritis: can anti‐cytokine therapy play a role in treatment? Clin Rheumatol 2010; 29: 451–455. 20108016 10.1007/s10067-009-1352-3

[6] Hunter DJ . Pharmacologic therapy for osteoarthritis–the era of disease modification. Nat Rev Rheumatol 2011; 7: 13–22. 21079644 10.1038/nrrheum.2010.178

[7] Liew FY , Girard JP , Turnquist HR . Interleukin‐33 in health and disease. Nat Rev Immunol. 2016; 16: 676–689. 27640624 10.1038/nri.2016.95

[8] Liu X , Hammel M , He Y *et al* Structural insights into the interaction of IL‐33 with its receptors. Proc Natl Acad Sci USA 2013; 110: 14918–14923. 23980170 10.1073/pnas.1308651110 PMC3773798

[9] Liu X , Li M , Wu Y , Zhou Y , Zeng L , Huang T . Anti‐IL‐33 antibody treatment inhibits airway inflammation in a murine model of allergic asthma. Biochem Biophy Res Commun 2009; 386: 181–185. 10.1016/j.bbrc.2009.06.008 19508862

[10] Kim YH , Yang TY , Park CS *et al* Anti‐IL‐33 antibody has a therapeutic effect in a murine model of allergic rhinitis. Allergy Eur J Allergy Clin Immunol 2012; 67: 183–190. 10.1111/j.1398-9995.2011.02735.x 22050307

[11] Qiu C , Li Y , Li M *et al* Anti‐interleukin‐33 inhibits cigarette smoke‐induced lung inflammation in mice. Immunology 2013; 138: 76–82. 23078031 10.1111/imm.12020 PMC3533703

[12] Peng G , Mu Z , Cui L *et al* Anti‐IL‐33 antibody has a therapeutic effect in an atopic dermatitis murine model induced by 2, 4‐dinitrochlorobenzene. Inflammation 2018; 41: 154–163. 28952069 10.1007/s10753-017-0673-7

[13] Li C , Maillet I , Mackowiak C *et al* Experimental atopic dermatitis depends on IL‐33R signaling via MyD88 in dendritic cells. Cell Death Dis 2017; 8: e2735. 28383552 10.1038/cddis.2017.90 PMC5477596

[14] Akcay A , Nguyen Q , He Z *et al* IL‐33 exacerbates acute kidney injury. J Am Soc Nephrol: JASN 2011; 22: 2057–2067. 21949094 10.1681/ASN.2010091011 PMC3279998

[15] Park GH , Shinn HK , Kang JH , Na WJ , Kim YH , Park CS . Anti‐interleukin‐33 Reduces Ovalbumin‐Induced Nephrotoxicity and Expression of Kidney Injury Molecule‐1. Intl Neurourol J 2016; 20: 114–121. 10.5213/inj.1632578.289 PMC4932645 27377943

[16] Jiang HR , Milovanović M , Allan D *et al* IL‐33 attenuates EAE by suppressing IL‐17 and IFN‐γ production and inducing alternatively activated macrophages. Eur J Immunol 2012; 42: 1804–1814. 22585447 10.1002/eji.201141947

[17] Li M , Li Y , Liu X , Gao X , Wang Y . IL‐33 blockade suppresses the development of experimental autoimmune encephalomyelitis in C57BL/6 mice. J Neuroimmunol 2012; 247: 25–31. 22516472 10.1016/j.jneuroim.2012.03.016

[18] Matsuyama Y , Okazaki H , Tamemoto H *et al* Increased levels of interleukin 33 in sera and synovial fluid from patients with active rheumatoid arthritis. J Rheumatol 2010; 37: 18–25. 19918048 10.3899/jrheum.090492

[19] Hong YS , Moon SJ , Joo YB *et al* Measurement of interleukin‐33 (IL‐33) and IL‐33 receptors (sST2 and ST2L) in patients with rheumatoid arthritis. J Korean Med Sci 2011; 26: 1132–1139. 21935266 10.3346/jkms.2011.26.9.1132 PMC3172648

[20] Palmer G , Talabot‐Ayer D , Lamacchia C *et al* Inhibition of interleukin‐33 signaling attenuates the severity of experimental arthritis. Arthritis Rheum 2009; 60: 738–749. 19248109 10.1002/art.24305

[21] Bateman JF , Rowley L , Belluoccio D *et al* Transcriptomics of wild‐type mice and mice lacking ADAMTS‐5 activity identifies genes involved in osteoarthritis initiation and cartilage destruction. Arthritis Rheum 2013; 65: 1547–1560. 23436205 10.1002/art.37900

[22] Kung LHW , Ravi V , Rowley L , Bell KM , Little CB , Bateman JF . Comprehensive Expression Analysis of microRNAs and mRNAs in Synovial Tissue from a Mouse Model of Early Post‐Traumatic Osteoarthritis. Sci Rep 2017; 7: 17701. 29255152 10.1038/s41598-017-17545-1 PMC5735155

[23] Loeser RF , Olex AL , McNulty MA *et al* Microarray analysis reveals age‐related differences in gene expression during the development of osteoarthritis in mice. Arthritis Rheum 2012; 64: 705–717. 21972019 10.1002/art.33388 PMC3269534

[24] Goldring MB . Articular cartilage degradation in osteoarthritis. HSS J 2012; 8: 7–9. 23372517 10.1007/s11420-011-9250-z PMC3295961

[25] Li C , Chen K , Kang H *et al* Double‐stranded RNA released from damaged articular chondrocytes promotes cartilage degeneration via Toll‐like receptor 3‐interleukin‐33 pathway. Cell Death Dis 2017; 8: e3165. 29095435 10.1038/cddis.2017.534 PMC5775407

[26] Watt FE , Gulati M . New drug treatments for osteoarthritis: what is on the horizon? Eur Med Journal Rheumatol 2017; 2: 50–58. 30364878 PMC6198938

[27] Zheng B , Zhang Z , Black CM , de Crombrugghe B , Denton CP . Ligand‐dependent genetic recombination in fibroblasts : a potentially powerful technique for investigating gene function in fibrosis. Am J Pathol 2002; 160: 1609–1617. 12000713 10.1016/S0002-9440(10)61108-X PMC1850857

[28] Zarpelon AC , Cunha TM , Alves‐Filho JC *et al* IL‐33/ST2 signalling contributes to carrageenin‐induced innate inflammation and inflammatory pain: role of cytokines, endothelin‐1 and prostaglandin E2. Br J Pharmacol 2013; 169: 90–101. 23347081 10.1111/bph.12110 PMC3632241

[29] Glasson SS , Blanchet TJ , Morris EA . The surgical destabilization of the medial meniscus (DMM) model of osteoarthritis in the 129/SvEv mouse. Osteoarthr Cartil 2007; 15: 1061–1069. 10.1016/j.joca.2007.03.006 17470400

[30] Choi WS , Lee G , Song WH *et al* The CH25H‐CYP7B1‐RORα axis of cholesterol metabolism regulates osteoarthritis. Nature 2019; 566: 254–258. 30728500 10.1038/s41586-019-0920-1

[31] Glasson SS , Chambers MG , Van Den Berg WB , Little CB . The OARSI histopathology initiative ‐ recommendations for histological assessments of osteoarthritis in the mouse. Osteoarthr Cartil 2010; 18(Suppl 3): S17–23. 10.1016/j.joca.2010.05.025 20864019

[32] Krenn V , Morawietz L , Burmester GR *et al* Synovitis score: discrimination between chronic low‐grade and high‐grade synovitis. Histopathology 2006; 49: 358–364. 16978198 10.1111/j.1365-2559.2006.02508.x

[33] Little CB , Barai A , Burkhardt D *et al* Matrix metalloproteinase 13‐deficient mice are resistant to osteoarthritic cartilage erosion but not chondrocyte hypertrophy or osteophyte development. Arthritis Rheum 2009; 60: 3723–3733. 19950295 10.1002/art.25002 PMC2832925

[34] Zhao J , Ouyang Q , Hu Z *et al* A protocol for the culture and isolation of murine synovial fibroblasts. Biomed Reports 2016; 5: 171–175. 10.3892/br.2016.708 PMC4950553 27446536

