# Supplementary material for: Blockade of IL‐33 signalling attenuates osteoarthritis
Source: Clin Transl Immunology. 2020 Oct 23;9(10):e1185. doi: 10.1002/cti2.1187 (PMC7587452; doi:10.1002/cti2.1187)
Supplement: Supplementary file 1 [file CTI2-9-e1185-s001.docx]

**
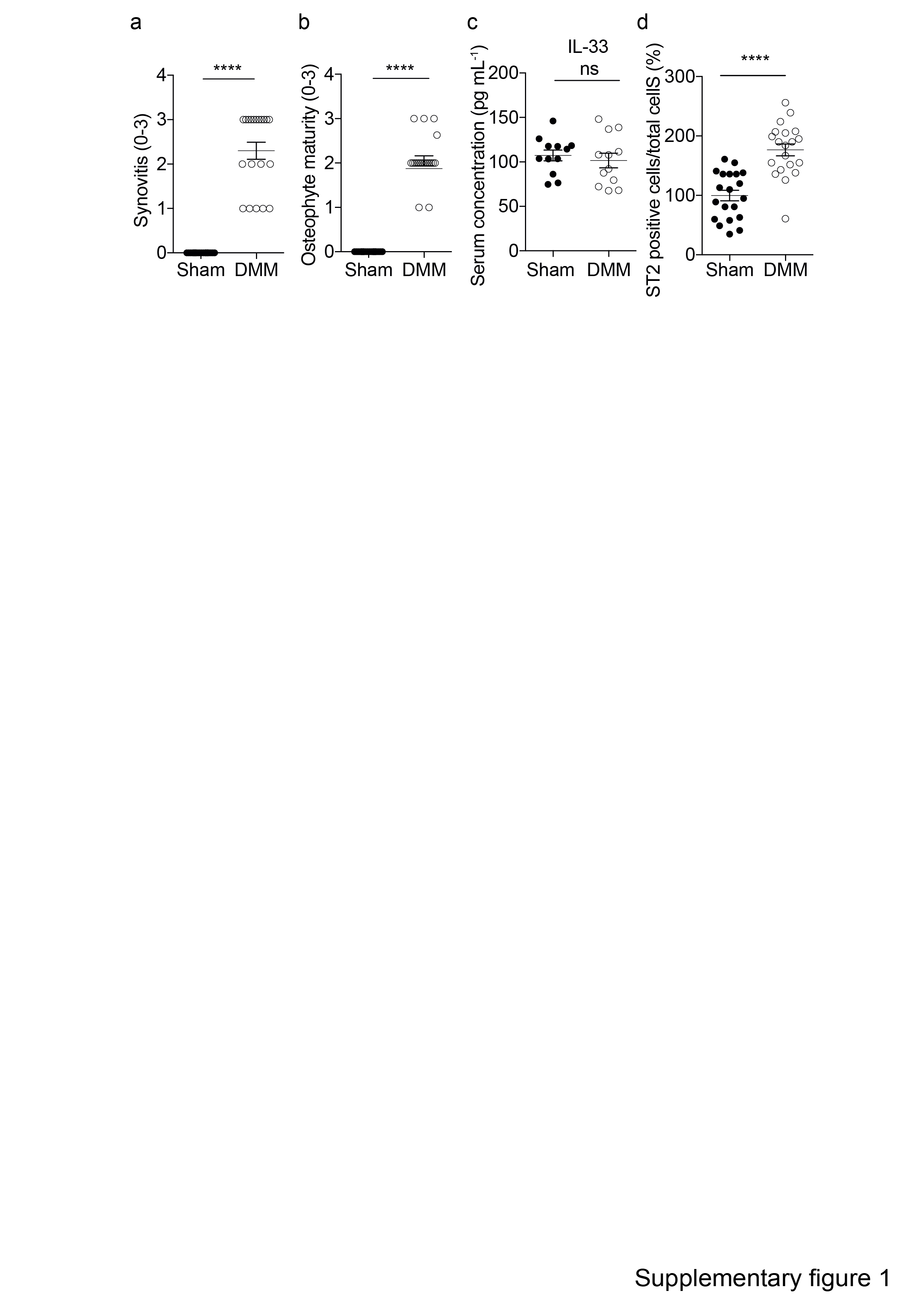
**

**Supplementary figure 1: Disease scoring and IL-33 and ST2 expression in murine OA. (a)** synovitis scoring and **(b)** osteophyte maturity scoring of sham- (*n*=20) or DMM- (*n*=20) operated WT mice (12 weeks post-surgery end timepoint). **(c)** IL-33 concentration (pg mL ^-1^) in serum of sham- (*n*=12) or DMM- (*n*=12) operated WT mice (12 weeks post-surgery end timepoint). **(d)** Quantification of ST2 positive cells/total from knee joints of sham- (*n*=20) or DMM- (*n*=20) operated WT mice (12 weeks post-surgery end timepoint). All RT-qPCR gene expressions were normalized to the endogenous level of 18s in respective groups. Data are expressed as mean ± S.E.M with with unpaired 2-tailed Student’s *t*-tests. *n* indicates the number of human specimens or mice per group. NS= non-significant. *P* < 0.0001 represented as ****.


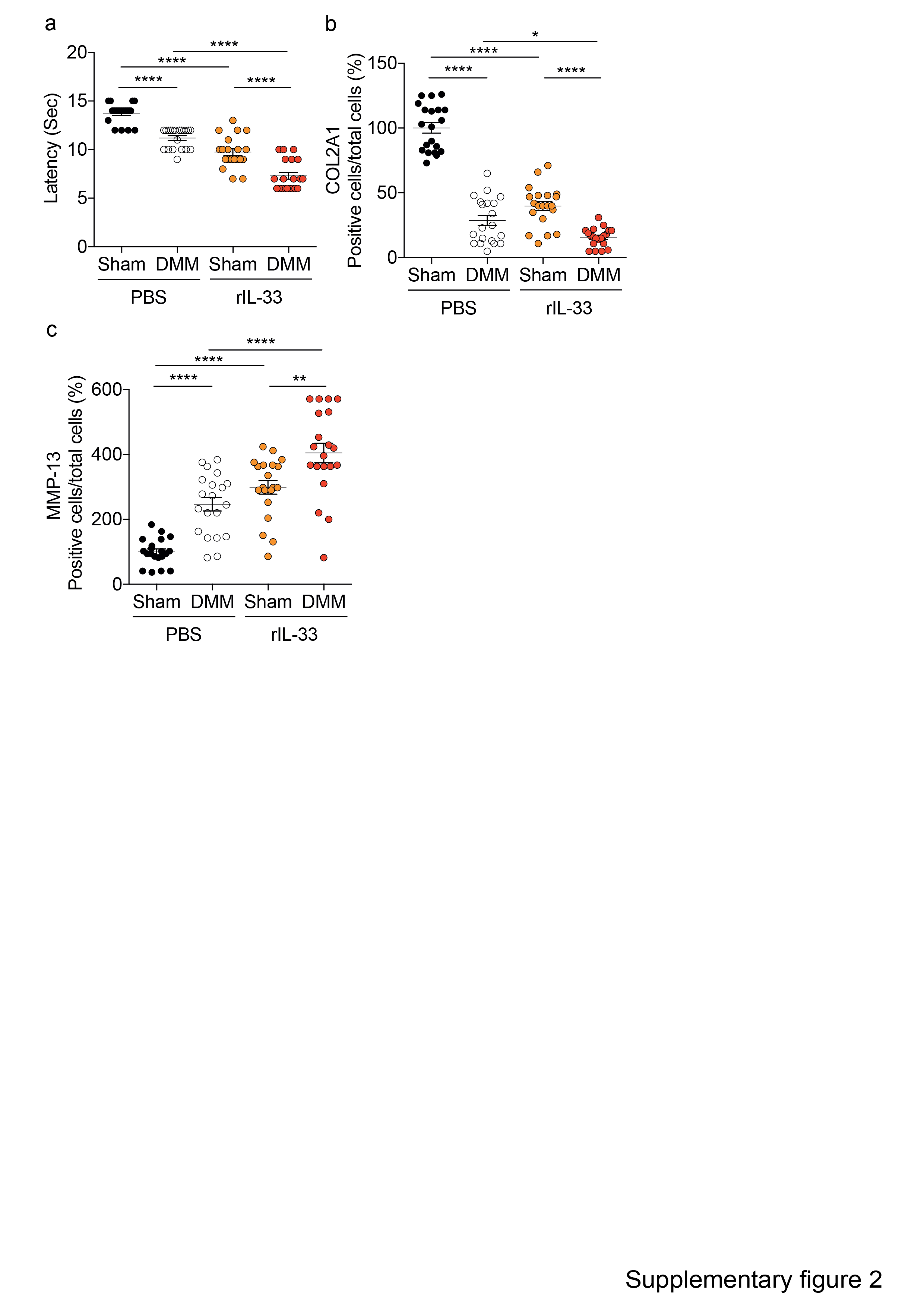


**Supplementary figure 2: Assessment of pain and OA markers in mice with increased IL-33 levels 12 weeks post DMM surgery. (a)** Hot plate pain assay of sham- (*n*=20) or DMM- (*n*=20) operated WT mice (12 weeks post-surgery end timepoint) treated intraperitoneally with either PBS (vehicle control) or rIL-33 (33 μg/kg; daily for 12 weeks post-surgery). Quantification of **(b)** COL2A1 and **(c)** MMP-13 positive cells/total from knee joints of sham- (*n*=20) or DMM- (*n*=20) operated WT mice (12 weeks post-surgery end timepoint) treated intraperitoneally with either PBS (vehicle control) or rIL-33 (33 μg/kg; daily for 12 weeks post-surgery). Data are expressed as mean ± S.E.M with two-way analysis of variance followed by the Tukey-Kramer test (a-c). *n* indicates the number of human specimens or mice per group. NS= non-significant. *P* < 0.05, *P* < 0.01 or *P* < 0.0001 represented as *, ** or **** respectively.


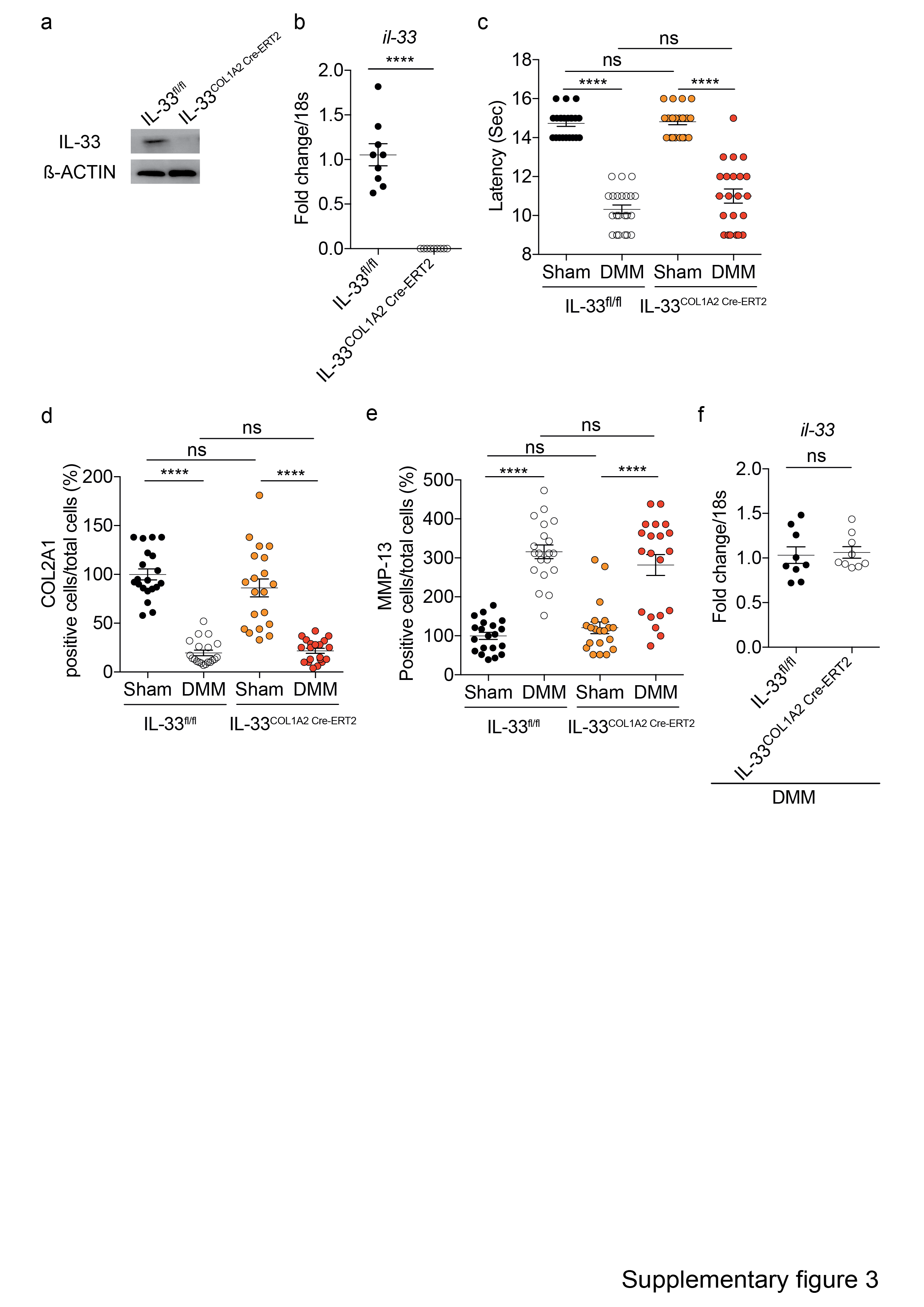


**Supplementary figure 3: Validation of IL-33^COL1A2 Cre-ERT2^ mice and determination of pain and OA markers in IL-33^COL1A2 Cre-ERT2^ mice 12 weeks post DMM surgery. (a)** Protein expression of IL-33 in synovium obtained from (10 weeks old) IL-33^COL1A2 Cre-ERT2^ and IL-33^fl/fl^ control mice. **(b)** mRNA expression in synovium obtained from IL-33^COL1A2 Cre-ERT2^ mice and IL-33^fl/fl^ control mice (10 weeks old mice; *n*=9). **(c)** Hot plate pain assay of sham- (*n*=20) or DMM- (*n*=20) operated IL-33^fl/fl^ control mice and IL-33^COL1A2 Cre-ERT2^ mice (12 weeks post-surgery end timepoint). **(d)** COL2A1 and **(e)** MMP-13 positive cells/total from knee joints of sham- (*n*=20) or DMM- (*n*=20) operated IL-33^fl/fl^ control mice and IL-33^COL1A2 Cre-ERT2^ mice (12 weeks post-surgery end timepoint). **(f)** mRNA expression of IL-33 in whole knee joints of DMM- (*n*=9) operated IL-33^fl/fl^ control mice and IL-33^COL1A2 Cre-ERT2^ mice (12 weeks post-surgery end timepoint). All RT-qPCR gene expressions were normalized to the endogenous level of 18s in respective groups. Data are expressed as mean ± S.E.M with two-way analysis of variance followed by the Tukey-Kramer test (c-e) or with unpaired 2-tailed Student’s *t*-tests (b, f). *n* indicates the number of human specimens or mice per group. NS= non-significant. *P* < 0.0001 represented as ****.

**
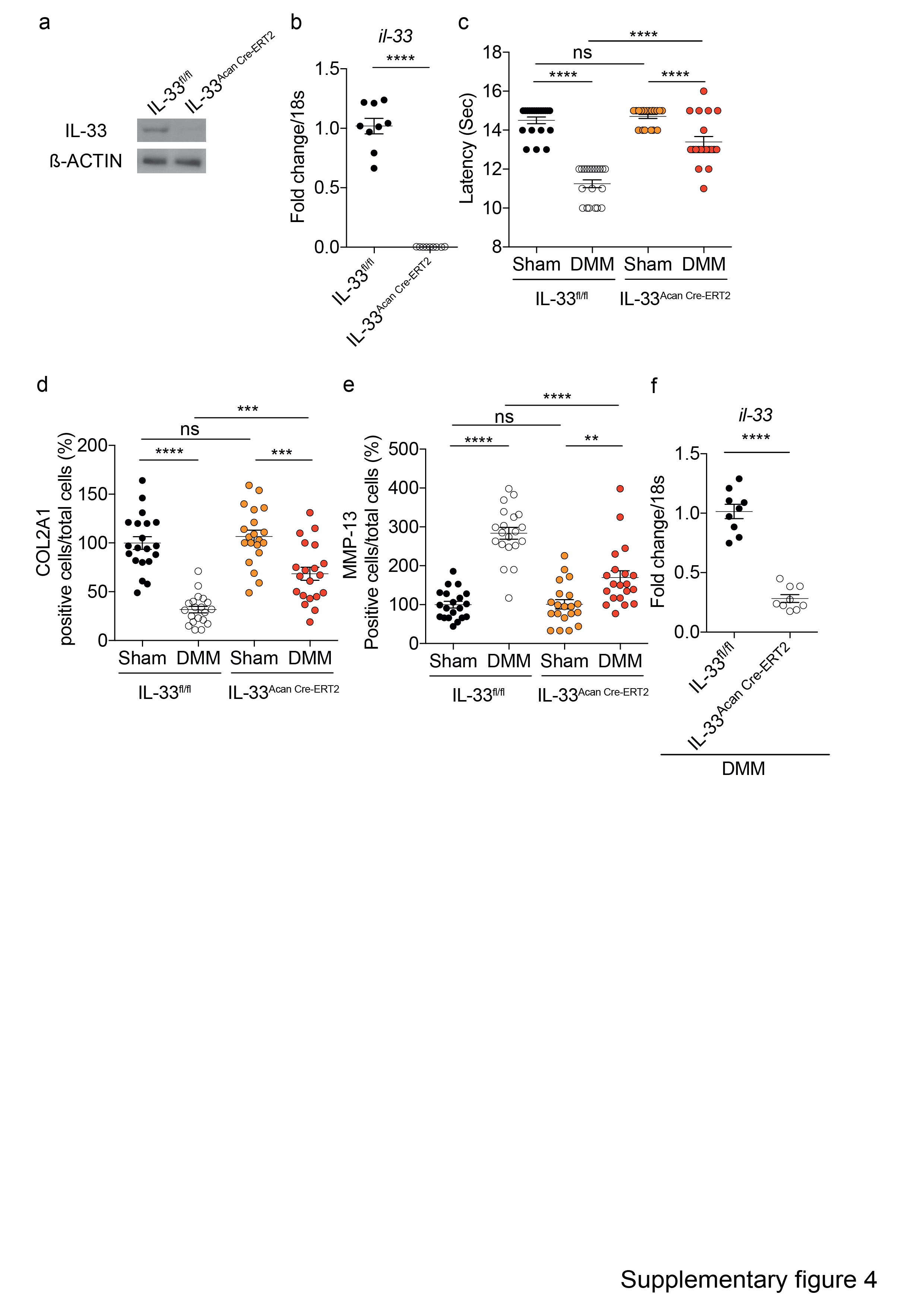
**

**Supplementary figure 4: Validation of IL-33^Acan Cre-ERT2^ mice and determination of pain and OA markers in IL-33^Acan Cre-ERT2^ mice 12 weeks post DMM surgery. (a)** Protein expression of IL-33 from cartilaginous hips avulsed from young (7 weeks old) IL-33^Acan Cre-ERT2^ and IL-33^fl/fl^ control mice. **(b)** mRNA expression in microdissected articular cartilage from knees joints obtained from IL-33^Acan 2 Cre-ERT2^ mice and IL-33^fl/fl^ control mice (10 weeks old mice; *n*=9). **(c)** Hot plate pain assay of sham- (*n*=20) or DMM- (*n*=20) operated IL-33^fl/fl^ control mice and IL-33^Acan Cre-ERT2^ mice (12 weeks post-surgery end timepoint). **(d)** COL2A1 and **(e)** MMP-13 positive cells/total from knee joints of sham- (*n*=20) or DMM- (*n*=20) operated IL-33^fl/fl^ control mice and IL-33^Acan Cre-ERT2^ mice (12 weeks post-surgery end timepoint). **(f)** mRNA expression of IL-33 in whole knee joints of DMM- (*n*=9) operated IL-33^fl/fl^ control mice and IL-33^Acan Cre-ERT2^ mice (12 weeks post-surgery end timepoint). All RT-qPCR gene expressions were normalized to the endogenous level of 18s in respective groups. Data are expressed as mean ± S.E.M with two-way analysis of variance followed by the Tukey-Kramer test (c-e) or with unpaired 2-tailed Student’s *t*-tests (b, f). NS= non-significant. *P* < 0.01, *P* < 0.001 or *P* < 0.0001 represented as **, *** or **** respectively.

**
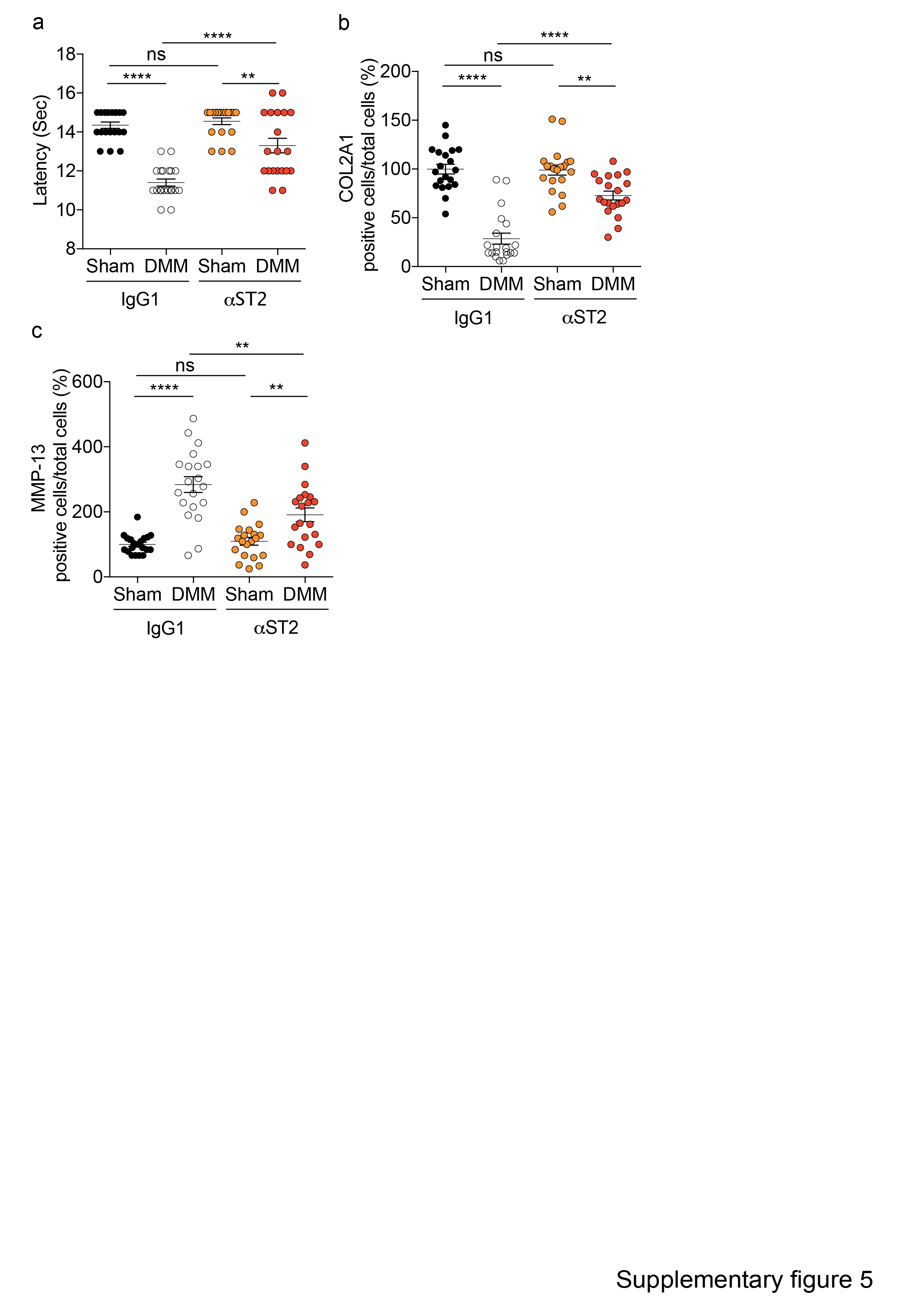
**

**Supplementary figure 5: Assessment of pain and OA markers in mice treated with αST2**

**12 weeks post DMM surgery. (a)** Hot plate pain assay of sham- (*n*=20) or DMM- (*n*=20) operated WT mice (12 weeks post-surgery end timepoint) treated intraperitoneally with either IgG1 (vehicle control; 50 µg per mouse; daily for 12 weeks post-surgery) or αST2(50 µg per mouse; daily for 12 weeks post-surgery). Quantification of **(b)** COL2A1 and **(c)** MMP-13 positive cells/total from knee joints of sham- (*n*=20) or DMM- (*n*=20) operated WT mice (12 weeks post-surgery end timepoint) treated intraperitoneally with either IgG1 (vehicle control; 50 µg per mouse; daily for 12 weeks post-surgery) or αST2 (50 µg per mouse; daily for 12 weeks post-surgery). Data are expressed as mean ± S.E.M with two-way analysis of variance followed by the Tukey-Kramer test (a-c). *n* indicates the number of human specimens or mice per group. NS= non-significant. *P* < 0.01 or *P* < 0.0001 represented as ** or **** respectively.


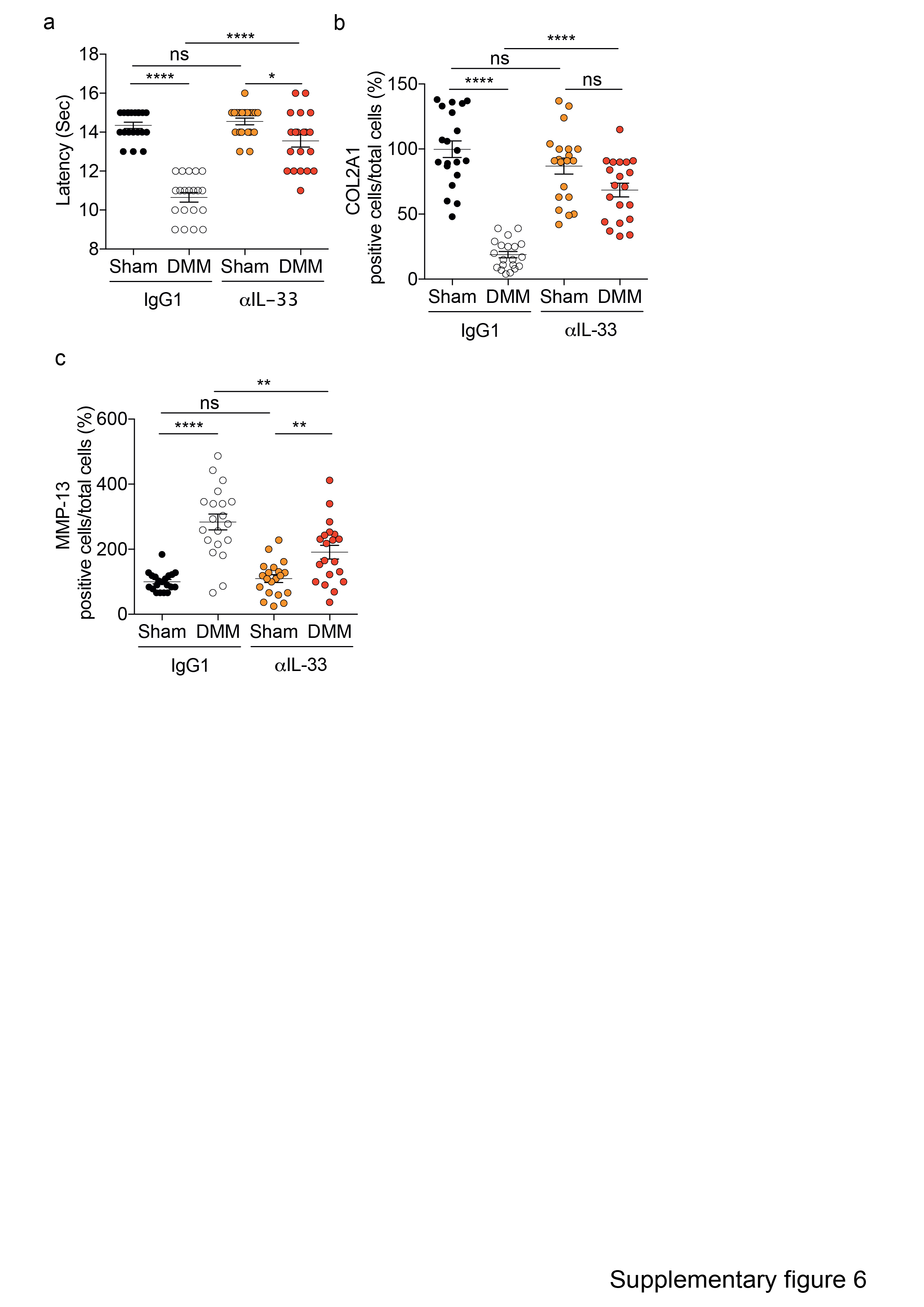


**Supplementary figure 6: Assessment of pain and OA markers in mice treated with αIL-33 12 weeks post DMM surgery. (a)** Hot plate pain assay of sham- (*n*=20) or DMM- (*n*=20) operated WT mice (12 weeks post-surgery end timepoint) treated intraperitoneally with either IgG1 (vehicle control; 15 µg per mouse; daily for 12 weeks post-surgery) or αIL-33 (15 µg per mouse; daily for 12 weeks post-surgery). Quantification of **(b)** COL2A1 and **(c)** MMP-13 positive cells/total from knee joints of sham- (*n*=20) or DMM- (*n*=20) operated WT mice (12 weeks post-surgery end timepoint) treated intraperitoneally with either IgG1 (vehicle control; 15 µg per mouse; daily for 12 weeks post-surgery) or αIL-33 (15 µg per mouse; daily for 12 weeks post-surgery). Data are expressed as mean ± S.E.M with two-way analysis of variance followed by the Tukey-Kramer test (a-c). *n* indicates the number of human specimens or mice per group. NS= non-significant. *P* < 0.05, *P* < 0.01 or *P* < 0.0001 represented as *, ** or **** respectively.

**Supplementary Table 1:** TaqMan probes used in RTq-PCR experiments

| **Gene** | **Human** | **Murine** |
| --- | --- | --- |
| *18S* | Hs03003631_g1 | Mm02601776_g1 |
| *ACAN* | Hs00153936_m1 | Mm00545794_m1 |
| *ADAMTS-5* | Hs00199841_m1 | Mm00478620_m1 |
| *COL2A1* | Hs00264051_m1 | Mm01309565_m1 |
| *IL-33* | Hs04931857_m1 | Mm00505403_m1 |
| *MMP-3* | Hs06633340_s1 | Mm00440296_g1 |
| *MMP-13* | Hs00233992_m1 | Mm00439491_m1 |
| *SOX-9* | Hs01001343_g1 | Mm00448840_m1 |
| *ST2* | Hs05059655_s1 | Mm00516117_m1 |
